# Reduced precipitation can induce ecosystem regime shifts in lakes by increasing internal nutrient recycling

**DOI:** 10.1038/s41598-024-62810-9

**Published:** 2024-05-30

**Authors:** Jordi Catalan, Agustín P. Monteoliva, José Carlos Vega, Almudena Domínguez, Ana I. Negro, Rocío Alonso, Blas Valero Garcés, Meritxell Batalla, Héctor García-Gómez, Manel Leira, Carlos Nuño, José Pahissa, María Peg, Sergi Pla-Rabés, Neftalí Roblas, José Luis Vargas, Manuel Toro

**Affiliations:** 1 CSIC, Bellaterra, Barcelona Spain; 2 CREAF, Cerdanyola del Vallés, Barcelona Spain; 3 ECOHYDROS, Camargo, Cantabria Spain; 4 Laboratorio de Limnología, Parque Natural del Lago de Sanabria y Alrededores, Rabanillo-Galende, Zamora Spain; 5 Centre for Hydrographic Studies, CEDEX, Madrid, Spain; 6 Area of Ecology, Faculty of Biology, University of Salamanca, Salamanca, Spain; 7 Ecotoxicology of Air Pollution, Environment Department, CIEMAT, Madrid, Spain; 8 Instituto Pirenaico de Ecología, CSIC, Zaragoza, Spain; 9 Department of Functional Biology, University of Santiago de Compostela, Santiago de Compostela, Spain; 10 Unitat Ecologia, BABVE, Universitat Autònoma de Barcelona, Cerdanyola del Vallés, Barcelona Spain

**Keywords:** Climate change, Ecosystem regime shift, Conditional heteroscedasticity, Precipitation decline, Global warming, Nutrient retention, Internal nutrient loading, Diatom blooms, Shifting reference states, Long-term monitoring, Lake Sanabria, Freshwater ecology, Limnology

## Abstract

Eutrophication is a main threat to continental aquatic ecosystems. Prevention and amelioration actions have been taken under the assumption of a stable climate, which needs reconsideration. Here, we show that reduced precipitation can bring a lake ecosystem to a more productive regime even with a decline in nutrient external load. By analyzing time series of several decades in the largest lake of the Iberian Peninsula, we found autocorrelated changes in the variance of state variables (i.e., chlorophyll and oxygen) indicative of a transient situation towards a new ecosystem regime. Indeed, exceptional planktonic diatom blooms have occurred during the last few years, and the sediment record shows a shift in phytoplankton composition and an increase in nutrient retention. Reduced precipitation almost doubled the water residence time in the lake, enhancing the relevance of internal processes. This study demonstrates that ecological quality targets for aquatic ecosystems must be tailored to the changing climatic conditions for appropriate stewardship.

## Introduction

Eutrophication has been the main worldwide concern for inland aquatic ecosystems during the last decades since the Great Acceleration^1,2^. Amelioration, restoration, and prevention actions have proliferated across countries as the problem was recognized: initially, with initiatives to reduce nutrient loadings^3^ and, progressively, developing sophisticated regulations to achieve sustainable water and ecosystem quality targets^4^. Climate change challenges these actions, formerly conceived under the assumption of a stable climate at human social scales^5^. The knowledge of climate and nutrient dynamics interactions is still limited, particularly concerning their combined influence as drivers of aquatic communities^6,7^. This understanding is becoming more urgent as climate change progresses and aquatic systems are affected across planet^8^. Anticipating ecosystem regime shifts is a socially urgent and scientifically challenging issue under currently shifting environmental conditions^9^. The climatically driven transition and eventual new situation could be socially perceived as a failure in water quality management without necessarily being the case. Traditional monitoring and new observation systems^10–12^ of lake key state variables may provide sufficient mid-term data to characterize and sometimes anticipate significant ecosystem transitions driven by climate shifts.

Any lake ecosystem shows a dynamic state (regime) with characteristic stochastic fluctuations and cycles resulting from internal feedback at the ecosystem scale in response to a hierarchical coupling with the external drivers at some characteristic time scales^13^. Lake communities reflect the ecosystem regime^14^. The two primary external drivers are climate forcing and nutrient loading from the catchment, each with their own dynamic regimes. Changes in external nutrient loading regimes have shown rapid lake responses, evidencing eutrophication and recovery within decadal scales^15–17^. On the other hand, paleolimnological records show examples of lake regime shifts at centennial to decadal scales coupled with major climatic transitions^14^. Nevertheless, whether relatively mild climatic shifts occurring over a few decades can drive a lake regime shift without increasing external nutrient loading, only modifying the internal lake dynamics, is unclear and difficult to evaluate exclusively based on sedimentary records.

Air temperature is increasing throughout the planet, and the focus on its impact on aquatic ecosystems has been mainly on warming effects^18,19^. Precipitation is usually considered in a more extended temporal framework than air temperature^20^. Undoubtedly, a large reduction in water inflow to a lake, affecting the water mass balance, will cause an extraordinary ecosystem shift^21^. However, in areas of relatively high precipitation, external nutrient loading may still be considered the main driver of the lake ecosystem state. Nevertheless, declining precipitation in these areas, although not altering lake level, may substantially modify lake water renewal, affecting nutrient retention, water column recycling, and internal loading from sediments, thus changing lake productivity and metabolism^22^. Consequently, it can be hypothesized that reduced precipitation could bring a lake ecosystem to a new, more productive regime, even with a decline in external nutrient loading. If the precipitation decline is incremental over decades, it may happen that the lake ecosystem experimented a gradual transition or an abrupt, rapid regime shift. The second case is of particular interest in the context of water quality management.

A regime shift is the process by which an ecosystem rapidly changes from one fundamental state to another. There is debate about the classification of these ecosystem rapid transitions concerning the mechanisms driving them^9,23^ and whether the regime shift can always be anticipated^23–25^. However, there is agreement that transitions should be indicated by ecosystem state variables showing a shift in their probability space, reflected in the autocorrelation patterns of their variance^26^. The critical aspect is the selection of those state variables and the appropriate temporal scale of observation and sampling frequency^27^. Increasing variance and autocorrelation in time series are early warnings of ecosystem shift^13^. In particular, conditional heteroscedasticity, which means that variance changes in an autocorrelated way, is a powerful indicator of regime shifts^28^. Variance in a time window step is not independent of the variance in the previous step. That is, periods of high variance are likely to follow periods of high variance, and conversely, those of low variance are more likely to be preceded by periods of low variance. Therefore, the potential non-linear effects of declining precipitation on lake ecosystems should be indicated by conditional heteroscedasticity in the time series of central ecosystem state variables such as chlorophyll, phytoplankton biomass, deep oxygen levels, or any other variable representative of the entire ecosystem metabolism. In the management context, when indications of significant conditional heteroscedasticity are found in monitoring time series of such key variables, the potential drivers have to be investigated to evaluate the mechanism that can be acting behind the abrupt change. Long-term monitoring of lakes has been extremely valuable for understanding eutrophication and the effectiveness of restoration actions by external nutrient loading reduction^29,30^. Nowadays, it can also provide the basis for a solid understanding of the effects of climate change^31^. Here, we describe a case study showing how a progressive decline in precipitation can produce an increase in lake productivity by enhancing the internal nutrient loading and shifting the ecosystem to a new regime. In the discussion, we consider the necessary general conditions required to increase lake productivity when the external nutrient load declines by enhancing the use efficiency and recycling of the nutrients.

**Figure 1:**
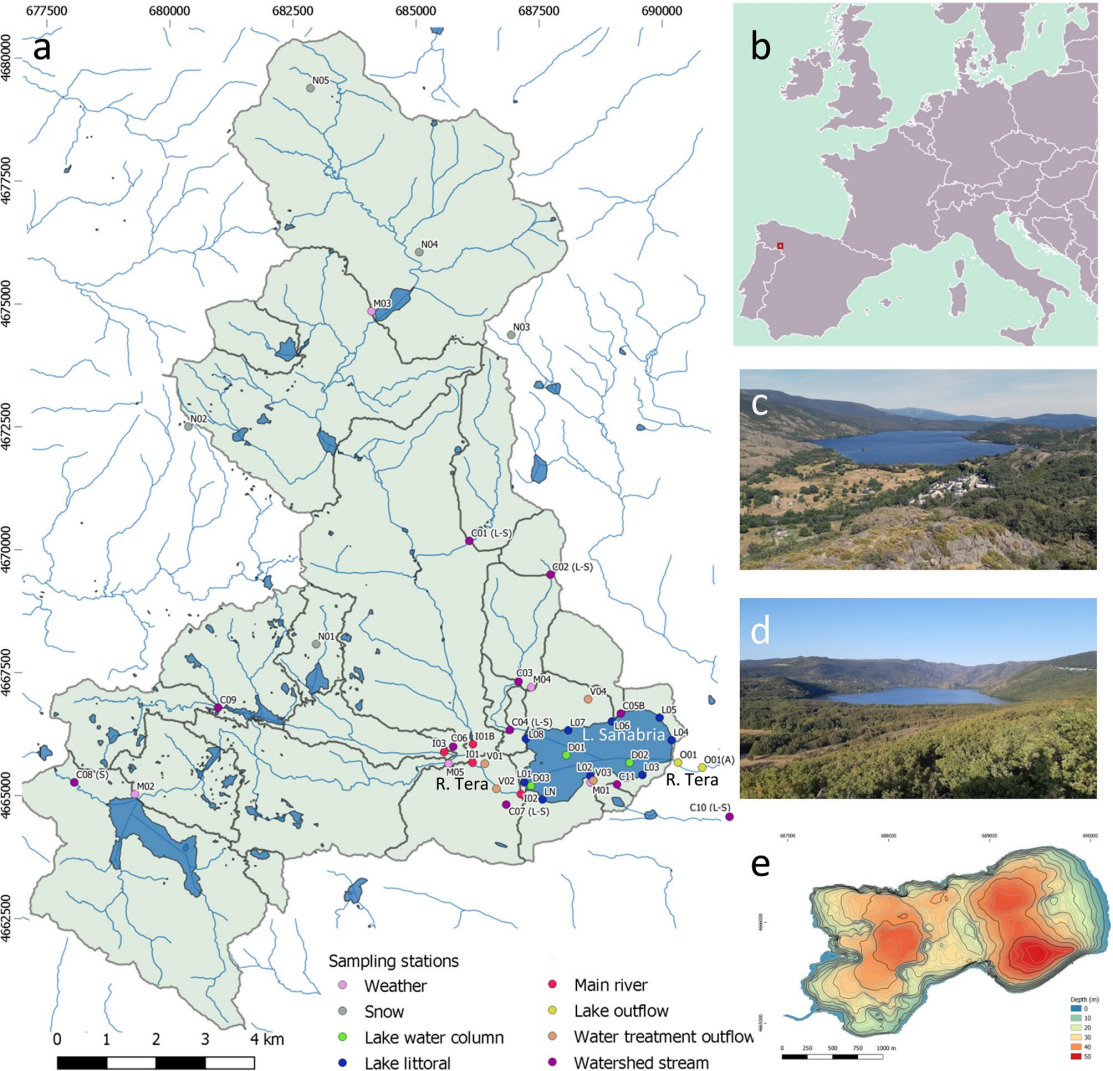
Lake Sanabria watershed. (**a**) Lake Sanabria basin, highlighting the sampling stations used in this study. Place names are indicated in Fig. S1. (**b**) Lake location within Europe. (**c**) Lake western, and (**d**) eastern views. (**e**) Lake bathymetry map, which can be found enlarged in Fig. S2.

Over thirty years of monthly monitoring in Lake Sanabria (northwestern Spain, 42°7′12.2″N, 6°42′27.9″W, 1004 m a.s.l, Fig. 1) have indicated a stable state in this oligotrophic lake until recently. The stability was not unexpected because the human population in its catchment is low; water treatment plants were built in the main villages during the 1990s; land use, primarily extensive livestock farming, progressively declined since the mid-twentieth century; and the atmospheric nitrogen deposition in the area has been historically low^32^. Surprisingly, sudden planktonic diatom blooms occurred in isolated years (2013 and 2017), embedded within apparently "normal" annual cycles. The blooms were of a single diatom species in each case (i.e., *Tabellaria flocculosa* and *Asterionella ralfsii* var *americana,* respectively). The puzzling situation raised social and scientific interest immediately after the first diatom proliferation, notably because Lake Sanabria is the largest natural lake in Spain. Since there was no evident proximate driver of the blooms, the question became whether the lake ecosystem was progressively changing to another state and for which reason.

The long-term time series of chlorophyll and oxygen measurements in Lake Sanabria—as primary indicators of lake metabolism—showed significant conditional heteroscedasticity earlier than the diatom blooms happened. The study of the sediment record showed a shift in the phytoplankton community, confirmed by comparing two years of intensive study with previous early phytoplankton records. However, the eventual drivers of the ecosystem regime shift were not evident. Therefore, we contrasted two main hypotheses: (1) increased diffuse external nutrient loading, perhaps related to the increasing afforestation of the catchment or the recreational use, or (2) increased internal nutrient loading and recycling within the lake basin, eventually related to changing climate conditions in the region^33^. The external nutrient loading trends were evaluated using (1) 30-year monthly nutrient data from the main inflow and (2) modeling external nutrient sources informed by a 2-year intensive sampling of the primary sources in the catchment (i.e., atmospheric deposition, livestock, recreation, runoff, erosion, subsurface flow, wastewater). Saharan dust intrusion events and occasional forest fires were also considered potential change drivers^34^. No evidence of external nutrient increase was found. Therefore, changes in internal nutrient use and recycling eventually linked to climate shift should be responsible for the lake regime shift. Weather decadal trends indicated a marked decline in precipitation, with no significant trend in average air temperature. The 30-year time series of lake monitoring showed an increase in the water retention time in the lake, which favored a more efficient use of nutrients in the water column, higher storage in the sediments, and conditions for nutrient return from the sediments. Overall, the Lake Sanabria case study provides evidence that progressive decline in precipitation driving incremental changes in water residence time may result in abrupt changes in lake productivity, resulting in a lake ecosystem regime shift.

## Results

### Lake metabolism regime shift

**Figure 2:**
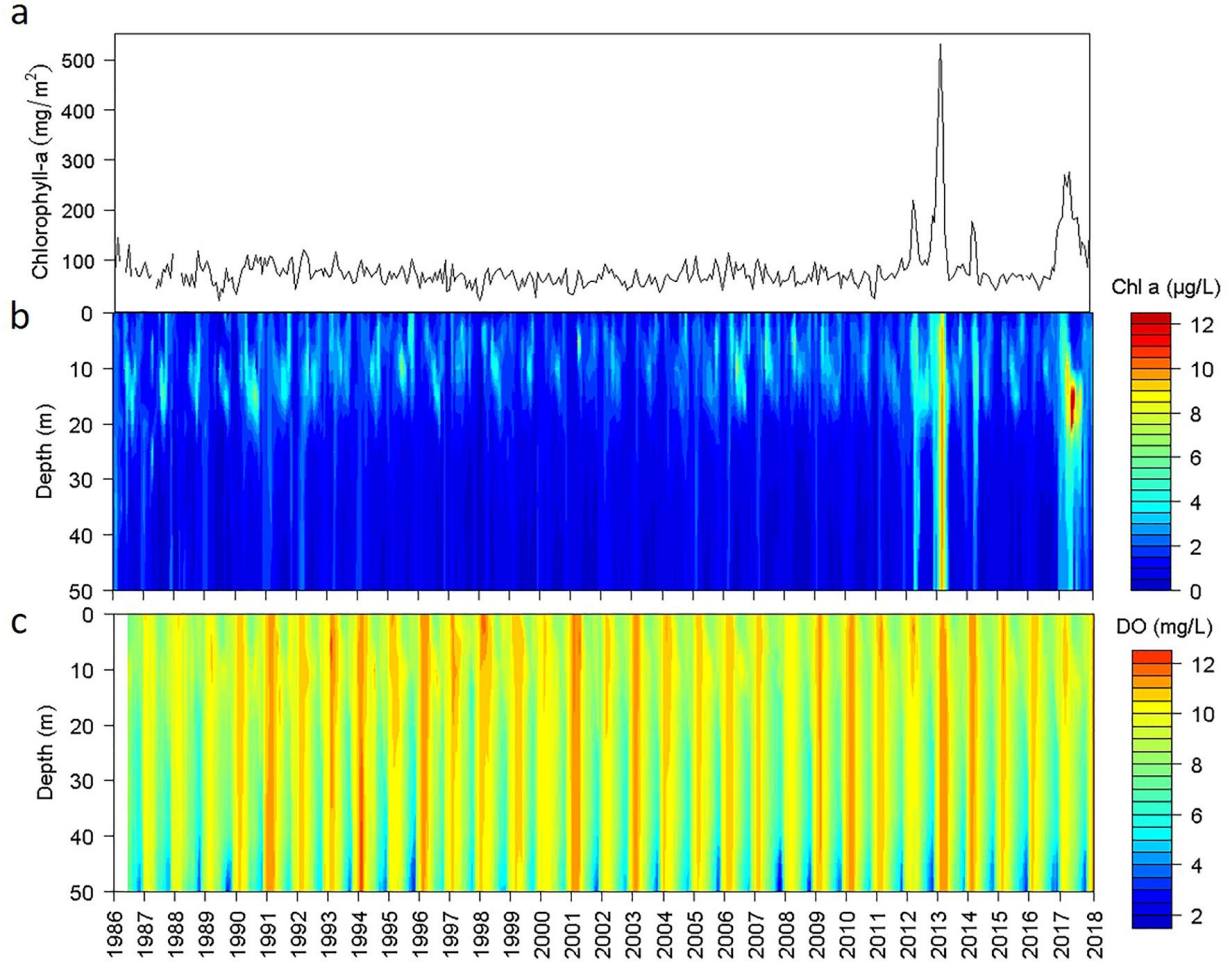
Time series (1986–2018) in Lake Sanabria of water column integrated chlorophyll (**a**), and chlorophyll (**b**), and oxygen (**c**) variation across depth, based on monthly measurements at every 2.5 m depth in the D02 station in the deepest part of the eastern basin.

**Figure 3:**
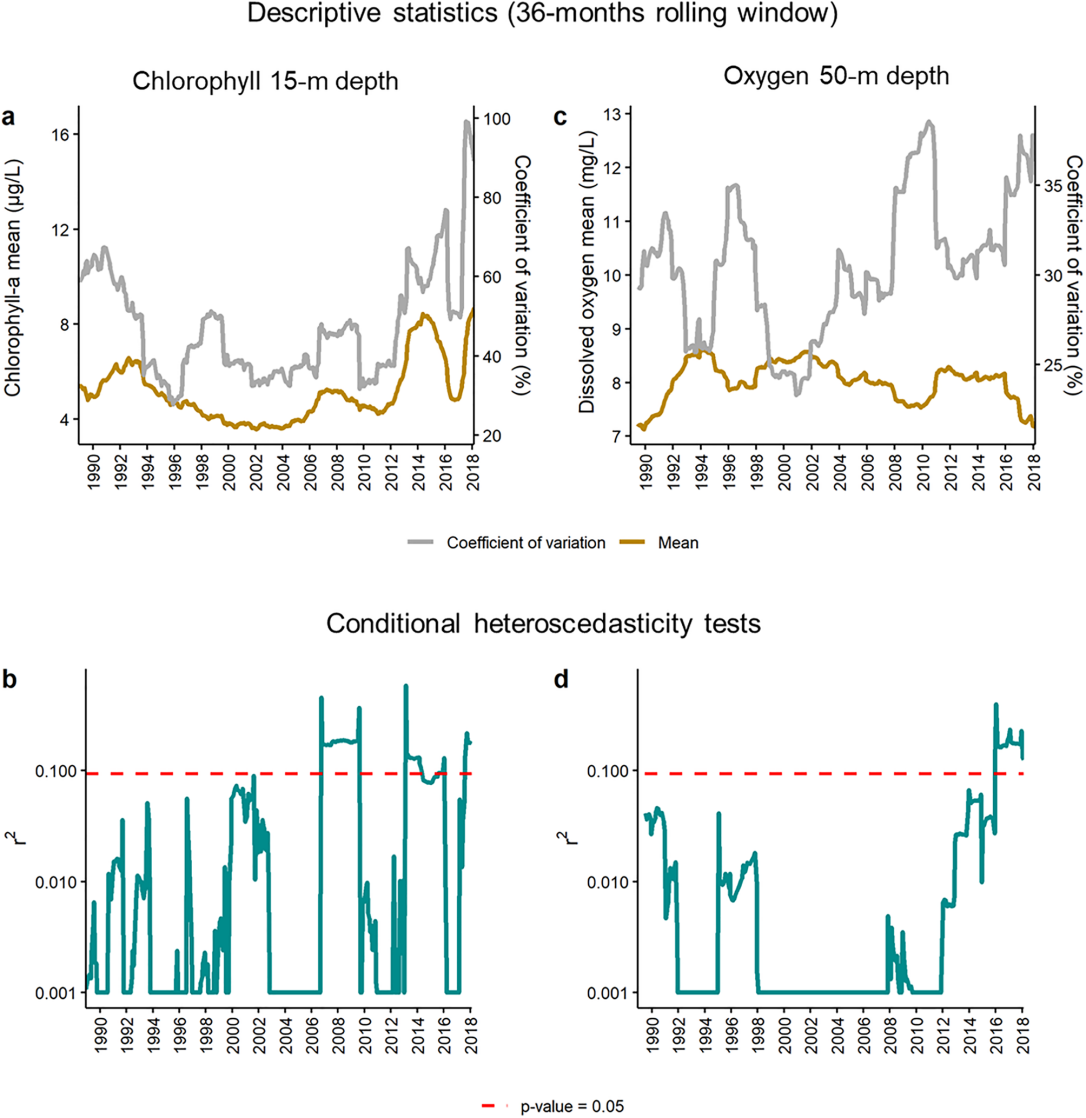
Changes in the mid-term statistics of chlorophyll at 15 m depth, where chlorophyll maxima usually occur, and oxygen at *ca.* 50 m depth, near the bottom of the deepest point. A 36-month rolling window was considered to estimate the statistical descriptors (**a**,**c**), and the conditional heteroscedasticity tests^28^ (**b**,**d**): values above the red line indicate significant conditional heteroscedasticity (*P* < 0.05).

The indicators of lake metabolism, such as chlorophyll and oxygen levels, have shown changing patterns of interannual variation during the last decade compared to the previous three decades of lake monitoring (Fig. 2). The water column-integrated chlorophyll achieved values several times higher than the usual long-term range—of 5 and threefold in 2013 and 2017, respectively. Chlorophyll maxima, which usually occurred relatively deep in the water column (12–20 m), also increased the concentration with similar ratios. The phytoplankton blooms were primarily due to a single but different diatom species in each period. For 2013, ad hoc surveys indicated that the bloom was due to *Tabellaria flocculosa,* and monthly assessments during 2017 showed that the proliferation was, in essence, of diatoms (Fig. S3), mostly *Asterionella ralfsii* var *americana* even though *Tabellaria* was also present (Fig. S4). The chlorophyll variation was within a narrow range for over two decades before 2013. From 1995 to 2005, there was a period of slightly lower chlorophyll values and higher water transparency (e.g., > 9 m Secchi disk depths were typical), but the chlorophyll oscillations were similar before and after this period. Although the chlorophyll mean and coefficient of variation did not start to change until 2013 (Fig. 3a), conditional heteroscedasticity showed a significant change earlier, towards 2007 (Fig. 3b), anticipating a potential regime shift.

The two exceptional algal blooms did not produce enhanced oxygen saturation within the photic zone, and they were also not apparent in oxygen depletion deeper in the water column. The oxygen time series were flatter than the chlorophyll series (Fig. 2). The only apparent feature in the series was an increase in the frequency of low oxygen years in the deepest layers during summer. Indeed, the time series of oxygen measurements near the bottom showed large fluctuations for the whole period (Fig. 2c). However, only recently (ca. 2016), the series started to show significant conditional heteroscedasticity (Fig. 3b), supporting the anticipation of a regime shift indicated by chlorophyll.

### External nutrient loads

The Tera River is the main inflow to the lake (91% of total modeled water inputs). Nutrient concentrations just before the river joins the lake suggest no increase in nutrient loading in recent years (Fig. S5), especially considering the inflow decline during the last decade (see below). In 1998, total phosphorus concentration declined when wastewater treatment plants were implemented in the main villages of the catchment, although dissolved phosphorus maintained similar values. Nitrate concentrations have been relatively stable; they did not decline after the early treatment plant implementation but did so following an improvement in plant management during the last decade. Occasional peaks of nitrate concentration, notably in 2006, were related to forest fires. However, these peaks showed no discernible response in the lake metabolism indicators. In summary, trends of nutrient loads by the main inflow showed an opposite direction to the increased metabolism experienced by the lake in recent times.

**Figure 4:**
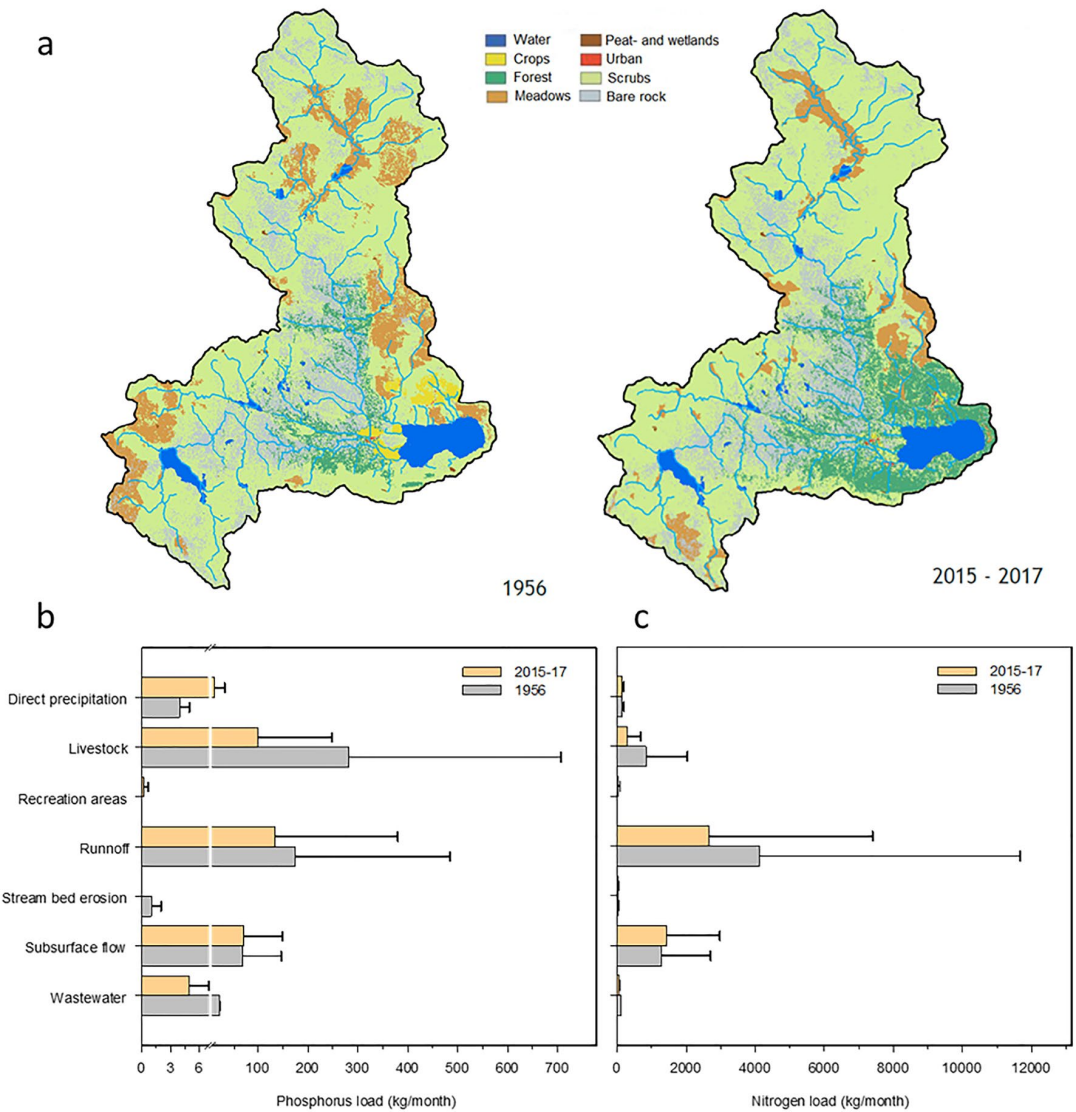
Contrasting land cover situations between 1956 and the present (**a**) in the Lake Sanabria watershed allows the comparison of phosphorus (**b**) and nitrogen (**c**) external load change according to the modeled contribution of primary sources.

There are no indications that small stream inflows and diffuse nutrient loads could be more relevant than direct Tera River inflows. Nevertheless, we performed an overall estimation of the nutrient loads to the lake considering land use, population, and recreational activities in the catchment, atmospheric deposition, and hydrological modeling of runoff and subsurface flows. The current main contribution is soil runoff for phosphorus and nitrogen (Fig. 4). Livestock was the main phosphorus contributor in the 1960s, and its influence has declined progressively since then. The reduction of this activity was associated with a landscape change. Oak forests increased, mainly around the lake, and following the main river course, meadows declined, and crops almost disappeared (Fig. 4a). The landscape change has resulted in a lower nutrient contribution from soils, with a more substantial decline in nitrogen. Although recreational use has recently increased during the summer period (pers. comm., Sanabria Natural Park, based on a vehicle gauging system), the overall human impact in the immediate zones around the lake has declined progressively because of decades of livestock and crop reduction. The current nutrient contribution by atmospheric deposition directly into the lake and catchment runoff is less than 10%. In conclusion, no evidence exists of external nutrient loading that could have fostered higher lake productivity and metabolism during the last decades.

### Climate and lake hydrodynamics shift

**Figure 5:**
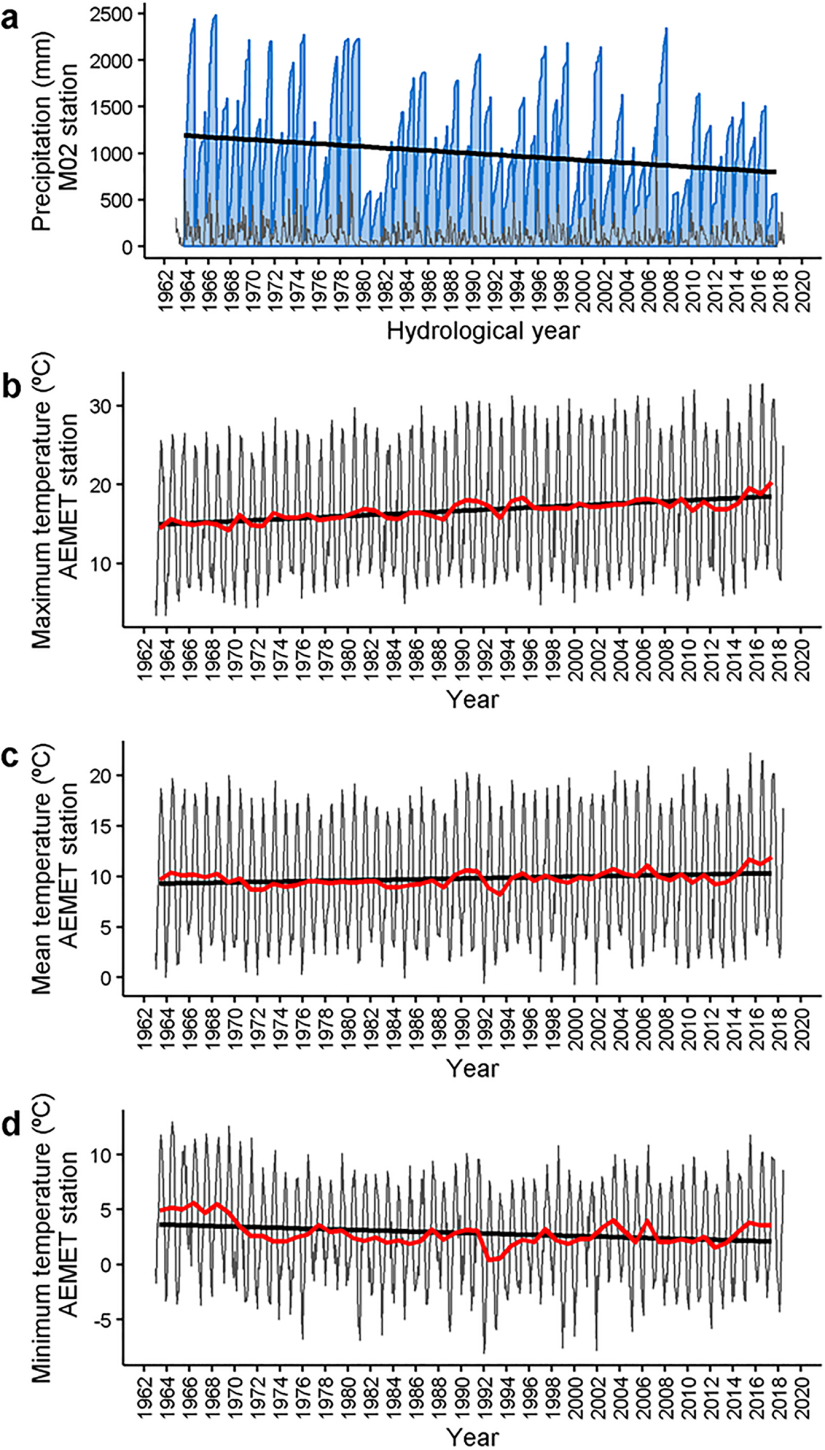
Weather trends in the Lake Sanabria area during the last decades. (**a**) Precipitation at Puente Porto (M02 station), and (**b**–**d**) temperature at Puebla de Sanabria (AEMET 2770B station). The latter is located outside the lake watershed but at a short distance. According to the Mann–Kendall trend test, linear tendencies (black line) are significant for precipitation (*P* = 0.001, n = 666), maximum (*P* = 0.0006, n = 666), and minimum temperature (*P* = 0.017, n = 666). The red line indicates a one-year moving average.

The most apparent climate shift in the Lake Sanabria watershed has been a decline in annual precipitation during the last decades (Fig. 5). For the period 1960–1980, annual values above 2000 L/m^2^ were common (~ 50%), and years below 1000 L/m^2^ were infrequent (< 10%). In contrast, during the last decade, annual precipitation rarely has achieved 1500 L/m^2^, and years with values barely above 500 L/m^2^ are common (30%). The monthly mean of the average daily temperature does not show any significant trend during the last fifty years (Fig. 5), although maximum (increasing) and minimum (slightly declining) temperatures show opposing significant trends.

**Figure 6:**
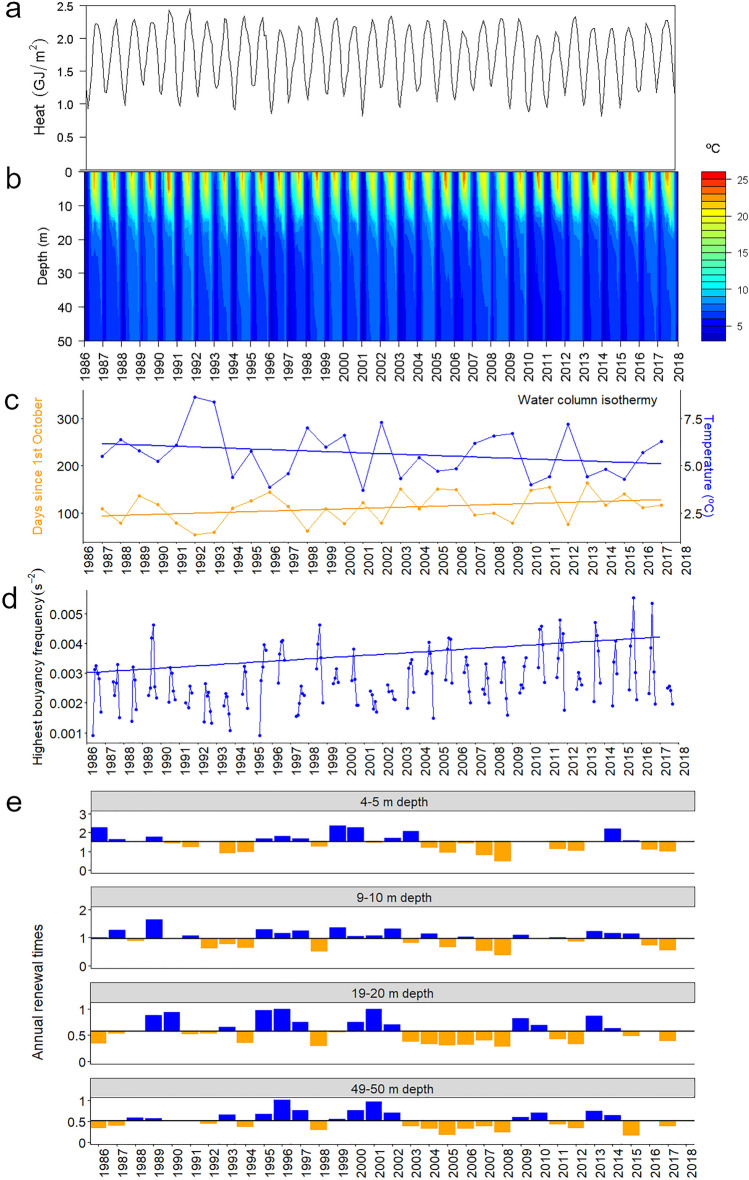
Lake Sanabria physical trends during the last decades. (**a**) Water column heat and (**b**) temperature patterns. (**c**) First date and temperature of lake isothermy (Mann–Kendall trend test, n = 31, *P* = 0.005, *P* = 0.0008, respectively). (**d**) Highest monthly buoyancy frequency during the stratification period (Mann–Kendall test, n = 32, *P* = 0.02). (**e**) Number of times that water renews annually for some selected depth layers across the water column. The black line indicates the average for 1986–2018 and bars above (blue) and below (orange) deviations.

These recorded climatic shifts should have had consequences in lake hydrodynamics. At first glance, the heat balance and water column thermal structure did not show remarkable changes (Fig. 6a,b). However, a more detailed consideration showed significant long-term trends in the holomixis patterns. The first date of isothermy was, on average, delayed, and the temperature of complete mixing declined about one degree (Fig. 6c). These changes were related to a stronger stratification indicated by a trend of increasing maximum buoyancy frequency (Fig. 6d) and a slight deepening of this maximum from about 8 to 10 m depth. The onset of the stratification has also been delayed. Consequently, the duration of stratification changed less than the shift in the starting and ending dates.

The water column physical changes and the inflow decline increased the lake's average water residence time (Fig. 6e). On average, layers around 10 m depth renew once per year, upper layers about twice yearly, and deep layers renewal time usually takes more than two years (Fig. 6e). However, there was a substantial difference around these average values before and after ca. 2003. Since then, renewal time has been significantly longer, particularly for layers below the seasonal thermocline (~ 10 m depth).

### Lake nutrient retention and recycling

During the last decades, external nutrient loading to the lake has declined mainly because of the recession in watershed livestock and the lower inflow. Conversely, the longer water residence time could have increased nutrient retention efficiency, bioavailability, and recycling—that is, internal loading. No direct experimental evidence of these processes was available, but from observations, there were several indicators of enhanced recycling: (a) the lower the Tera River flow, the higher the reduction between inlet and outlet total phosphorus concentration (Fig. S6a). The difference was significant for both stratification and mixing periods, although the reduction was more substantial during the former. (b) Nitrate concentration was systematically higher in the water column than at the lake inflow (Table 1), indicating organic nitrogen recycling and posterior nitrification. The nitrate formation was higher during stratification, and there was a noisy, albeit significant, correlation with phosphorus retention (Fig. S6b). (c) Organic matter was enriched in carbon the deeper the sediment traps were located (Fig. S7), and the difference between the epilimnetic trap (lower C/N) and the rest (higher C/N) was higher during stratification when the C/N ratio was lower, suggestive of higher algal contribution. Furthermore, layers below the photic zone showed two significant complementary trends (Fig. S8) that explain the progressive higher oxygen depletion observed (Fig. 2). On the one hand, oxygen consumption rates increased in deep layers, a few meters above sediments. On the other, layers 30 to 40 m depth did not show this tendency but prolonged their periods of oxygen decline during stratification. Overall, these trends indicate a context of enhanced nutrient recycling and diffusion from the sediments.

**Table 1:** Lake Sanabria climate, watershed, and lake characteristics.

	Mean	Median	SD	Min	Max	n
Climate						
Annual precipitation* (L/m^2^)	1486	1487	455	556	2405	55
Annual mean temperature** (°C)	9.8	9.6	0.7	8.2	11.9	55
Coldest month average temperature** (°C)	1.8	1.7	1.1	-0.7	4.2	55
Warmest month average temperature** (°C)	18.7	18.6	1.4	16.1	22.3	55
Watershed						
Drainage basin area (km^2^)	119.5	–	–	–	–	–
Drainage density (m^−1^)	8.2 × 10^–4^	–	–	–	–	–
Elevation (m)	1633	1666	234	1004	2125	4,861,230
Terrain slope (%)	16.1	13.2	12.1	0	78.4	4,861,227
Areal water contribution (L/m^2^/year)	1376	1254	304	1254	1722	10
River inlet***						
River inflow (hm^3^/month)	10.93	8.75	8.64	0.026	46.27	432
Soluble reactive phosphorus (µg/L)	3.60	1.50	12.19	0.10	180	386
Total phosphorus (µg/L)	14.32	9.90	24.97	1.10	370	327
Nitrate (µg/L)	31	25	30	1.5	332	365
Silica (mg/L)	1.5	1.5	0.29	0.7	2.6	365
Lake***						
River inflow (hm^3^/month)	10.93	8.75	8.64	0.026	46.27	432
Water temperature (°C)	7.9	7.0	3.6	3.2	26	8127
Secchi disk depth (m)	6.9	6.7	1.4	3.5	12.2	387
Conductivity (µS/cm)	12.7	12.6	1.5	8.6	18.3	8064
pH	6.4	6.4	0.37	5.2	8.4	7894
Dissolved oxygen (mg/L)	9.0	9.3	1.6	1.5	12.3	8022
Soluble reactive phosphorus (µg/L)	1.21	1.00	1.22	0	20.5	6826
Total phosphorus (µg/L)	6.14	6.0	1.75	1.0	16.7	6322
Nitrate (µg/L)	49	45	31	0	221	7643
Silica (mg/L)	1.5	1.5	0.18	0	2.4	7662
Chlorophyll ɑ (µg/L)	1.49	1.20	1.18	0	12.4	8090

*M02 station data (1963–2018).

**AEMET 2770B station data (1963–2018).

***Lake Sanabria monthly monitoring data (1986–2018).

### Sediment records

**Figure 7:**
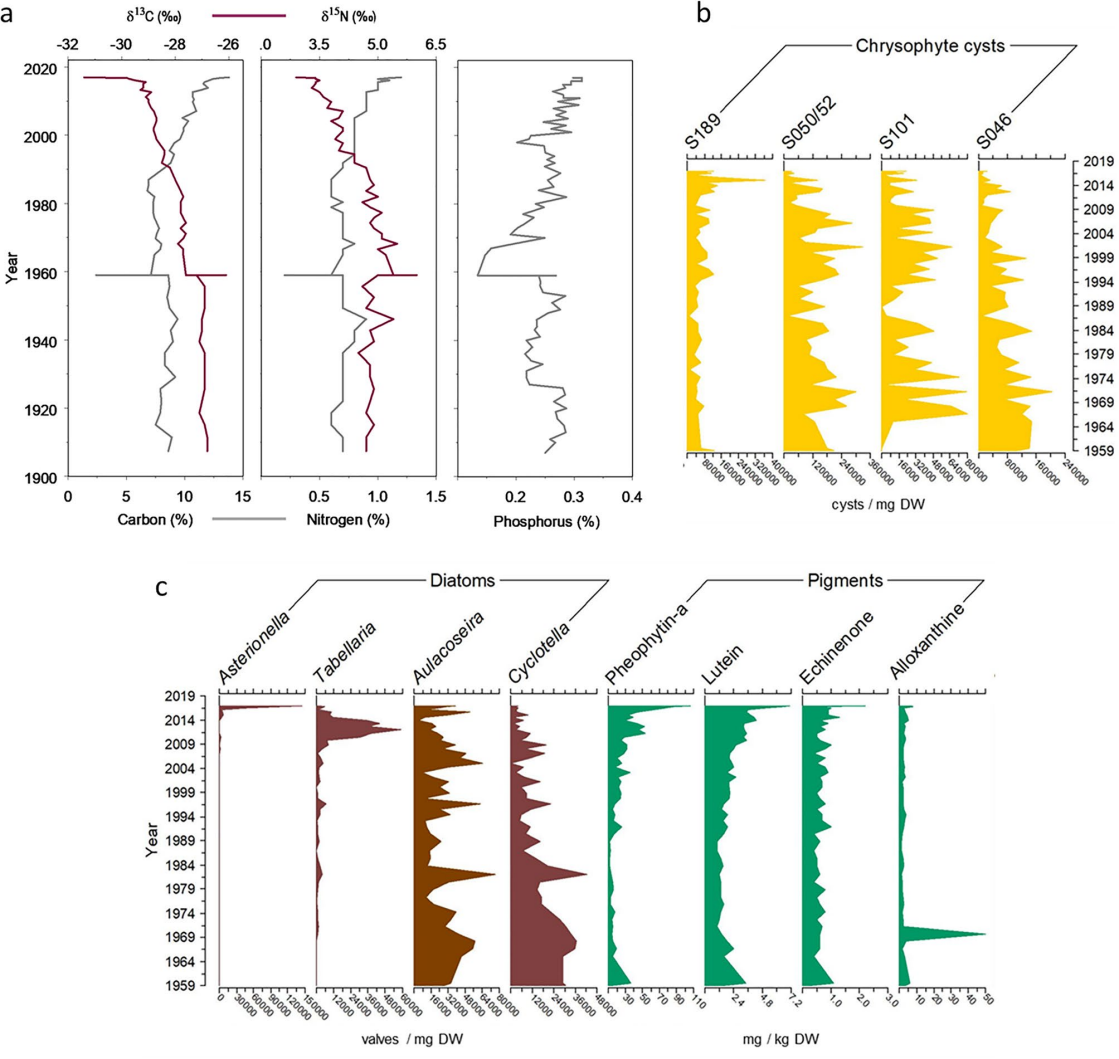
Sediment indicators of main functional and community changes in Lake Sanabria during the last decades (**a**) Organic carbon, total nitrogen, and total phosphorus sediment accumulation in the deepest part of the lake. Carbon and nitrogen stable isotopic compositions are also indicated (red line). Note the marked 1959 oscillation corresponding to the Vega de Tera dam collapse that reset the lake's biogeochemical dynamics. See Fig. S7 for the depth-age model. (**b**) Main chrysophyte cyst changes. (**c**) Diatom remains (*Asterionella ralfsii* var *americana*, *Tabellaria flocculosa, Aulacoseira subborealis/pseudodistans, and Cyclotella stelligera*), and representative algal pigments (pheophytin-a, bulk phytoplankton indicator; lutein, chlorophytes; echinenone, cyanobacteria; alloxanthin, cryptophytes).

The Vega de Tera dam failure about 10 km upstream of the lake on 9 January 1959 caused a flood that deposited a thick sandy-silt layer over the lake basin (Fig. S9). This event sealed the upper biogeochemically active sediment, causing a transient oligotrophication of the lake evidenced by the lower sediment carbon, nitrogen, and, particularly, phosphorus content during the following two decades (Fig. 7a). Similar conditions in phosphorus accumulation to those before the catastrophic event were achieved by the early 1980s. Since 2003, phosphorus accumulation maintained high values, with maxima during the last years. The sediments increased carbon and nitrogen content by the late 1980s, with a progressively more depleted signature in δ^13^C and δ^15^N, suggesting higher algal contribution. The P contrasting patterns with C and N during 1960–1990 indicated a progressive building of a P internal loading capacity.

Some key indicators summarize the main changes in the phytoplankton community since 1959 (Fig. 7b,c). Pheophytin-a indicates overall algal biomass; the values were fairly low until ~ 1990 and have risen since then. Lutein and echinenone, pigments respectively exclusive of chlorophytes and cyanobacteria, followed the same trend. Alloxanthin, a cryptophytes' indicator, also increased recently, confirmed by α-carotene (not shown), another pigment related to cryptophytes. Alloxanthin also showed high values, indicative of cryptophyte blooms, during the decade following the dam failure. The general patterns in the sediment record agreed with two periods of direct phytoplankton observations in the water column. Comparison between 1987 and 1989^35^ and our study 2015–2017 monthly observations (Fig. S10) indicated a decline in flagellated groups (chrysophytes, cryptophytes, and dinoflagellates) and an increase in chlorophytes, xanthophytes, and cyanobacteria.

Diatom valves and chrysophyte cysts in the sediments indicate highly fluctuating populations (Fig. 7b,c). Nonetheless, some species have declined (e.g., *Cyclotella stelligera,* cysts S046, S101, S050/52) during the period in which the pheophytin-a was increasing. Conversely, a few species markedly peaked during the last decade. *T. flocculosa* was the most conspicuous, and, more recently, *A. ralfsii*, both observed in the water column survey. Chrysophyte cyst S189 has increased since the 1990s and recently peaked. During the 2015–2017 study, we found this cyst within colonies of *Stichogloea doederleinii*, a coccal phaeothammniaceae species, which commonly peaks towards the end of the stratification period. The temporal stratification displacement may favor the *Stichogloea*-associated chrysophyte that has not yet been identified.

In summary, the sediment record indicates an increasing nutrient export to the sediments and a shifting phytoplankton community towards assemblages indicative of slightly higher productivity or, at least, different productivity patterns.

## Discussion

Eutrophication symptoms in lake ecosystems are usually associated with increasing external nutrient loads^36^. In Lake Sanabria, this has not been the case. On the contrary, nutrient loads were lower during the last decades because of livestock decline, forest expansion, improved wastewater treatment, and reduced river flow. Atmospheric deposition represents a minimal contribution of nutrients, and aerosol analyses indicated a low influence of anthropogenic activities in the area^34^. Conversely, there were indications that the increased lake productivity was related to a higher internal nutrient load, exemplifying the complex interplay between physical and biological mechanisms^37^.

We found that lower river flows resulted in higher nutrient retention efficiency and cycling processes, such as nitrification in the water column. Furthermore, progressively declining flushing rates during the last decades increased carbon, nitrogen, and phosphorus accumulation rates in the sediments that, associated with higher oxygen consumption rates during stratification, favor the nutrient diffusion from sediments, modifying the feedbacks of the biogeochemical dynamics of the lake^38^. Considering the small C/N sediment range during the last decades, indicative of low variability in terrestrial vegetation inputs, the recent δ^13^C decrease in Sanabria was likely attributed to higher lake primary productivity^39,40^, as also indicated by the shape of the P upper sediment profile^41^. The post-1980 decreasing trend in δ^15^N was coherent with higher productivity in a non-limited N environment facilitated by a higher internal sediment recycling^42^. Therefore, the lake's shifting trophic conditions were ultimately related to climate and the hierarchical links with proximate drivers^43^.

Climate is a multi-variate primary driver shaping the probability space in which the lake ecosystem oscillates^44^. If the regional climate changes, the mechanistic cascading from climate to the lake is affected, and, as a consequence, the stationary lake regime may be modified^45^. A regime shift is generally understood as a relatively sharp (non-linear) ecosystem change compared to more progressive (linear) trends in the drivers^46^. In Lake Sanabria, the climatic shift mainly consists of a persistent decline in precipitation during the last decades, which has been associated with the North Atlantic Oscillation and Scandinavian atmospheric circulation modes^47^. The declining flushing rates did not apparently produce lake ecosystem changes until the sudden diatom blooms. However, the analysis of variation of chlorophyll already anticipated that the transition started before.

Chlorophyll and oxygen concentrations are simple but powerful and complementary variables to characterize the lake's metabolic state. The chlorophyll conditional heteroscedasticity test anticipated the later more conspicuous changes in Lake Sanabria. The shift indicator was significant in 2007, whereas diatom blooms in the water column appeared in 2013 and 2017. Oxygen in deep layers showed a delayed response, which makes sense in a variable that integrates more extended periods. The sediment record confirmed a reorganization of the phytoplankton community beyond the apparent peaks of the two diatom species. The shifts in the sediment indicators were apparent by 2008, again before the blooms.

Sanabria changes in the lake community were most conspicuous in a few species, but sediment pigment records indicate that other groups change in abundance. Historical data for short periods and occasional annual samples^52^ compared to the biannual intensive recent study are inconclusive, beyond the diatom case, because of the high species richness and seasonal fluctuations. Nevertheless, provided the significant changes in relative dominance at high taxonomic levels (Fig. S10), a relevant shift in species composition is very likely.

We may wonder if the lake is shifting to a new regime without precedents or is switching to a state that has been present before. Indeed, Sanabria's Holocene sediment records show periods of high *Tabellaria flocculosa* accumulation, the latter about 3200 years ago, which lasted for ~ 800 years^48^. There were other similar periods in the Mid and Early Holocene. Interestingly, past *T. flocculosa* peaks occurred when geochemical and sedimentological indicators point to lower precipitation^48^. Therefore, we might hypothesize that the lake is shifting to a state already visited long ago. However, the temporal scales between the Holocene sediment record and the recent changes are substantially different, warning against using past fluctuations as direct analogs of the current situation.

Even in the case of similar past situations, it would not mean that the lake should oscillate between two alternative equilibria and, thus, be in a critical transition situation at present^49^. Some criteria have to be fulfilled to infer that a regime shift involves a critical transition^23^: (1) an abrupt shift in the time series has to occur, (2) driven by positive feedback mechanisms, (3) in response to an incremental increase in control parameters, which (4) results in a bimodal distribution of state variables, and (5) displays hysteresis in the transitions^50^. Some of these requirements are accomplished in the L. Sanabria case if it is assumed that the control parameter is the water renewal time and the feedback relies on nutrient cycling. Bimodality is incipient because the period of high chlorophyll values is shorter than the period of low, so at present, there is only a fat cue in the high values of the probability density function of the chlorophyll record. Hysteresis would require a more extended time series for an accurate test, with some back-and-forth oscillation between low and high precipitation years. Indeed, there is much debate about whether bimodality and the alternative state concept help to explain long-term patterns in lakes, even in the shallow lakes where the theory was inspired^51^, the temporal scale of observation being critical. The conditional heteroscedasticity test of state variables is a valuable operational option for anticipating an abrupt shift in lake ecosystems^26^. Understanding the limnological, environmental, and climatic mechanisms involved will provide the cues for adequate management.

The case of Lake Sanabria demonstrates that increased internal nutrient loading resulting from longer water residence time in a lake can drive an ecosystem regime shift towards a more productive state, even in cases with declining total external nutrient loading because of reduced precipitation. To generalize, a deliberately simple model of nutrient cycling within a lake (Appendix S1) helps to understand that reduced precipitation could more likely increase productivity in lake ecosystems when (1) the relative decline in external loading is lower than the precipitation decline, that is, nutrient concentrations in the inflow are maintained or increase; (2) there is an increase in the efficiency of the nutrient loading conversion to living biomass within the water column; for instance, by increasing the time for phosphatase action^53^, mineralization of organic nitrogen^54^ or recycling of refractory materials^55^, and (3) by enhancing exchanges with the sediments^56^ because of longer water residence time, favoring higher sedimenting biomass proportions, or more extended periods of nutrient release to the water column. Not all these requirements must be fulfilled, but they must be met in adequate relative proportions (Appendix S1). In the end, what is relevant is the relative (not absolute) shift in the recycling processes with respect to the previous state. Consequently, those lakes with initial low productivity yield of the external nutrient load or relatively low internal recycling will be more prompt to significant changes by reduced precipitation in their watersheds.

In Lake Sanabria, a large part of P and N loading is not in the form of simple inorganic compounds readily assimilable. Therefore, the increased residence time of water may enhance the use of a nutrient fraction that otherwise will circulate through the system in high-flow conditions. In other lakes, inorganic forms of limiting nutrients may be more relevant in the external loading and, thus, have less capacity to compensate for the decline in external loading by increasing assimilation efficiency^57^.

On the other hand, longer water residence time enhances the fraction of water column biomass that can sink to sediments and be recycled. A large proportion of sedimented P is in insoluble fractions, and only some (i.e., metal oxides) will release exchangeable P under reduced conditions^58^. The release will increase the longer the stratification period and the higher the oxygen demand in deep waters, as in Lake Sanabria. In Lake Sanabria, the water residence time during rainy periods was already much higher than the characteristic time for biomass sedimentation (Appendix S1). Consequently, the lake acted, already in the past, as an efficient trap with a limited margin for increase, and the sediment record reflected lake productivity. The enhanced exchange between water and sediment should not be restricted to deep waters; warming and reduced oxygen may happen across the benthic system, enhancing nutrient release and algae production^56^. Lakes experiencing more extended changes in water column stratification than Lake Sanabria may show a more substantial contribution by sediment nutrient release^38^.

The documented Lake Sanabria transition and the eventual new situation could be socially perceived as a failure in water quality management, particularly if blooms reach the surface, as happened in 2013. Many European countries have adopted the concept of reference conditions to determine the ecological states of their aquatic ecosystems, lakes in particular^59^. The reference conditions for a good ecological state are established under the assumption that lakes without human influence maintain communities and biogeochemical processes within a stationary range of oscillations driven by climate. Operatively, the concept was implemented by defining reference sites with low human impact, which provided an acceptable range of variation for lakes within the same category. The statistics could come from time series in those sites or from a statistical population of lakes considered to be of the same functional category^4^. Lake Sanabria was defined as unique in its category and became its own reference site, provided the human impact in the watershed was low^52^. Under changing climate, lake reference conditions will require overall revision, especially regarding productivity indicators such as phytoplankton and chlorophyll. Lake Sanabria provides a paradigmatic example of the difficulties in defining shifting reference conditions because climate change and the lake transition may last for decades and move towards no analog scenarios^60^. It could happen that there is not a shift from a stationary state to another but a permanent nonstationary state.

Consequently, authorities and administrations should shift from managing the state to managing change. Stewardship measures should complement, occasionally replace, conservation and restoration targets^61^. Those measures require understanding the drivers cascading from global climate to within lake processes and developing management safe-operating spaces to avoid undesirable watershed human actions that may synergistically interfere with shifting climate effects in unwanted ways. Non-stationarity in climate demands new theoretical and operative ways to deal with ecological dynamics^62^. The Lake Sanabria case demonstrates that ecological quality targets of aquatic ecosystems must be tailored to the new climatic conditions, even in localities with no remarkable water quality pressures. This study shows that precipitation decline in rainy regions can bring some lake ecosystems to the edge of regime shifts by modifying seasonal hydrodynamics, nutrient recycling, and internal loading.

## Methods

### Study site

Lake Sanabria (42°7′12.2″N, 6°42′27.9″W) constitutes a unique ecosystem among the inland waters of Spain, being the largest natural lake in the Iberian Peninsula (area 3.536 km^2^, volume 99.114 hm^3^). The lake has a relatively small watershed (~ 122.16 km^2^) (Fig. 1, S1), mainly of granitic bedrock, with the highest altitude at 2127 m a.s.l. (Peña Trevinca Peak) and the lake at 1004 m a.s.l. The climate is temperate, with precipitation distributed throughout the year but lower during the warm summer, showing high interannual variability (Table 1). The Tera River constitutes the main inflow, which since the late 1950s has been affected by the discharge of the hydroelectric power system of Moncabril in one of its main tributaries. In addition, small streams and underwater springs are discharging in the lake. Oak forests and meadows associated with small villages constitute the dominant vegetation around the lake. Above 1500 m a.s.l., scrublands, mountain pastures, and significant areas of peat bogs are the predominant categories. Lake Sanabria morphology is the result of glacier over-excavation at the bottom of a U-shaped cross-section valley closed at its eastern end by a terminal moraine^63^. The lake presents two sub-basins (Fig. 1e, S10): the western basin, receiving the Tera discharge, is slightly shallower (43 m) than the eastern (50 m), with the outlet. Almost 70% of the lake volume is above 20 m depth and ~ 10% below 30 m; this last volume is exposed to high contact with fine bottom sediments. The lake thermal regime is typically holomictic, with stratification from April to December. The lake has been experiencing epilimnetic autumn warming and hypolimnetic annual cooling during the last decades^33^. The temperature range in the upper layers is wide (Table 1), with epilimnetic waters > 20 °C during the summer months. According to the bedrock characteristics, ionic strength and acid-neutralizing capacity are low, and pH is circumneutral (Table 1). Chlorophyll, nutrient levels, and transparency indicate an oligotrophic state (Table 1), also reflected in the oxygen levels, which do not achieve elevated over-saturation nor anoxia in the water column. At the end of the stratification period, oxygen minima (~ 20–30% saturation) occur in the lake deepest part. Dissolved organic carbon (DOC) levels are relatively high for temperate mountain lakes (2–3 mg C L^−1^) because of the forested catchment and some peatlands in the upper catchment. The lake holds remarkable species-rich phytoplankton, zooplankton, and littoral communities^64^.

### Meteorological data

There are several running meteorological stations in the lake watershed (Fig. 1). The longest data series, covering the 1963–2018 period, were in Puente Porto reservoir (M02 station, 42°7′1″N, 6°49′52″W, 1645 m a.s.l), concerning precipitation, and Puebla de Sanabria (AEMET 2770B station, 42°3′15″N, 6°38′2″W, 960 m a.s.l), concerning temperature. The latter is located outside the lake watershed but not far away and shows high coherence with those in the lake's catchment. Gaps in these two meteorological series were filled using nearby stations and the Climatol R package (v 3.1.2)^65^. For air temperature, there is a high coherence among the regional stations. We used nine extra stations (including M02) to fill the gaps in the AEMET 2770B time series, which showed 22% of missing data. The adjusted R^2^ between the AEMET 2770B time series and the others was high (mean and median 0.83, min. 0.75, max. 0.9). The spatial coherence for precipitation is lower than for air temperature. However, the number of missing data was very low (7%), and the M02 station is located in the central part of the lake's watershed. We used 85 stations in a 150-km radius from the lake to fill the gaps. The adjusted R^2^ between the MO2 station and the other stations was uneven (adj. R^2^ median 0.62, min. 0.13, max. 0.80). The significance of the observed trends was assessed using the Mann–Kendall trend test.

### Lake long-term dynamics

Long-term lake monitoring (1986–2018) included monthly inlet and outlet samples and profiles in the eastern sub-basin (station D02) for temperature, conductivity, dissolved oxygen, Secchi disk depth, soluble reactive phosphorus (SRP), total phosphorus (TP), nitrate, silica, and chlorophyll. There was a gap in nitrogen and phosphorus data for some months of 2012 and 2013 due to laboratory equipment problems. Water samples were collected at 2.5 m intervals from the surface to 50 m depth, and the same lab made measurements throughout the period. Additionally, there was a period (2015–2017) of more comprehensive sampling at both sub-basins, including phytoplankton samples and littoral points.

Nitrate and SRP were determined by ion chromatography (940 Professional IC Vario TWO/SeS/PP Metrohm following EPA 300.1 method); total nitrogen (TN) and TP using segmented continuous microflow AutoAnalizer AA3; silica by spectrophotometry (V650 Jasc Standard Methods 4500-SiO2 C Ed 22); and DOC by high-temperature catalytic combustion (Formacs NC Analyzer with Nondispersive infrared detector (NDIR), Skalar). Chlorophyll was determined using Whatman® GF/F fiberglass filters, acetone extraction (90%), and spectrometry, and it was calculated according to Jeffrey and Humphrey^66^. Phytoplankton samples were fixed with Lugol's solution and counted with a Nikon Eclipse Ti-S inverted microscope following Utermöhl's method. Counting and biovolume determination followed European standards (EN 15204:2006, EN 16695:2015). Taxa were identified under 1000 × light microscopy, and, for diatoms, scanning electron microscopy was additionally used.

The water column's physical structure was considered by calculating the buoyancy frequency (N^2^) from the monthly temperature and conductivity profiles,

$${N}^{2}= \frac{-g}{{\rho }_{0}}\frac{\partial {\rho }_{z}}{{\partial }_{z}}$$

where g is the acceleration due to gravity, ρ is the water density, and z indicates depth. We derived some variables from N^2^ data to obtain time series that could reflect long-term changes in the lake water column physical dynamics: (a) the isothermy date, determined as the day with the lowest water column Schmidt stability^67^; (b) temperature at water column isothermy; (c) highest N^2^ during stratification; and (d) the depth where the highest N^2^ occurs.

The inflow density indicated that the river mixed with lake water above the thermocline during the stratification period. Therefore, assuming that the epilimnion (and the whole water column during lake isothermy) was mixed and the outflow was exclusively through the lake surface outlet of Tera River, we estimated the number of times each 1 m layer volume was renewed annually. In-lake phosphorus retention was estimated monthly from inlet and outlet concentrations and river flow.

During the stratification, the oxygen consumption in layers below 30 m depth was characterized by two parameters: (1) the duration of the layer isolation, considering the number of days between annual maximum and minimum oxygen values at the measurement depth, and (2) the rate of consumption, estimated from the slope of the regression of the oxygen measurements made during this period against time.

### Evaluation of changes in catchment nutrient sources

During the last decades, land use and human activities in the catchment have changed markedly. To evaluate whether potential nutrient loads could have increased, we modeled the nutrient loads delivered by the most relevant sources (i.e., direct precipitation, livestock, recreation areas, runoff, stream bed erosion, subsurface flow, and wastewater) for two scenarios corresponding to 1956, for which aerial images and land cover maps were available, and the period of intensive study (2015–2017), which provided information for the model adjustment. The lake's water inflow and nutrient loads were assessed by dynamic modeling on a 5 m spatial resolution digital elevation model^68^ validated with the intensive biennial sampling of catchment tributaries and atmospheric deposition. The hydrological model was calibrated and validated in two years of contrasting precipitation, 1620 L/m^2^ in 2016 and 1010 L/m^2^ in 2017. In each of these years, about 65% of the data was used for calibration, and the remaining 35% was used for validation. The nutrient export model was calibrated by land cover information and measurements at different points of the catchments (Fig. 1) and validated using nutrient data (a total of 21) from the river Tera at the lake inlet.

The watershed and stream network river delineation was computed using TauDEM (Terrain analysis using digital elevation models) tools^69^, implemented in QGIS-v2.8^70^. The river network was subsequently adapted to the reality of the terrain by checking on orthophotos and maps (i.e., channel rooting) and field observations (i.e., minimum order of the active channel). A total of 26 discontinuity points were established considering hydro-morphological and land use discontinuities: stream confluences, diversions and canals, reservoirs, primary land use typologies, and the main (Tera River) and secondary watercourses draining into the lake. The contribution areas to these points define basins or hydrologic response units, with areas ranging from approximately 20 ha to a maximum of 2500 ha.

The hydrological and nutrient load modeling was performed using MapShed (v1.5.1)^71^, which is a GIS-based tool that incorporates the generalized watershed loading functions (GWLF) model and enhances the functionality using a free GIS software package (MapWindow). MapShed combines hydrology, land cover, soils, topography, weather, pollutant discharges, and other data to determine the amount of daily nutrient and sediment loads in small watersheds. It is considered a combined distributed/aggregated catchment model and has been successfully applied in rural watershed studies^72^. The model simulates surface runoff using the curve number equation^73^ based on daily precipitation and temperature data and distinguishes between direct runoff and infiltration based on rainfall, soil, and land cover data. For each contributing area, a daily water balance was made based on precipitation, snowmelt, initial state and capacity of the unsaturated zone, and evapotranspiration. Streamflow consisted of total watershed runoff from all source areas plus groundwater discharge from a shallow saturated zone, also connected with a deep saturated zone (aquifer). Both fluxes were modelled by means of recession coefficients. It was assumed that there was no deep seepage connection with a regional aquifer system consistent with the glacial character of the basin. The solid fraction contribution was estimated as the product of the monthly sediment load and the average sediment nutrient concentration. Erosion was estimated using the universal soil loss equation (USLE algorithm), which used monthly values of the soil erodibility, topographic, cover and management, and soil and water conservation coefficients for each generating area, i.e., each combination of land use/cover and soil type. The sediment delivery ratio, the critical factor for computing sediment yield, was based on the watershed size and transport capacity.

Dissolved nutrient load and sediment transport through rural areas were computed by multiplying their respective coefficients by runoff. In the GWLF, all the N and P from the urban areas were considered to be in a solid state, and the model used exponential accumulation and wash-off function to estimate urban loading. The sub-surface losses in the watershed were estimated using dissolved N and P concentrations, where the watershed was considered a single lumped-parameter contributing area. The 5-cm upper soil was sampled and chemically analyzed twice (winter and summer) in six plots in catchment areas representative of the main land uses (i.e., grassland, scrubland, oak forest, and pastures with low and medium cattle pressure). The soil water content was measured and chemically analyzed at these plots, and an extra one was located in a peatland area at 20 and 40 cm depths for one year. These measurements were used to calibrate the soil and hydro-chemical module for erosion, runoff, and subsurface flows.

Livestock farming and urban wastewater contributions were accounted for in each lake watershed basin. Livestock comprised beef cattle and sheep in a seasonal grazing mode and was considered in the "open land" areas where it was known to be present. The model estimates contributions by basin using numbers and weight for each animal type. Nitrogen and phosphorus produced by farm animal populations are transported to nearby water bodies as losses due to animal grazing. The transport includes runoff from grazing land and direct deposits to streams where access is unimpeded.

The nitrogen and phosphorus loads from point sources were estimated considering two wastewater effluents from unitary sewage treatment systems, with direct discharge to the river Tera, 100 and 600 m upstream of the lake, and another discharging to the terrain at a distance 100 m to the lake coast. They were secondary treatments with an activated sludge process. Following Evans and Corradini^71^, a default nutrient retention factor was applied to the total exported loads to those basins with reservoirs as outlets.

Although the GWLF model could be used without calibration^74^, a tailored calibration technique for some relevant parameters and specific values for the Sanabria watershed was applied (Table S1). The model performance was evaluated by comparison with measurements at the river Tera inlet using data from November 2015 to September 2017, daily for discharge and monthly for nutrient concentration (Table S2). The agreement with the observed data was satisfactory (Fig. S11). For instance, the Nash–Sutcliffe efficiency statistics (NSE) showed values of 0.61 for river discharge, 0.64 for TP concentration, and 0.61 for TN (Table S2, Fig. S11). Generally, the simulation is considered satisfactory for this model when NSE > 0.5^75^.

The parameterized model was applied to two different periods: the year 1956 and the intensive sampling period (2015–2017), with different scenarios regarding weather, land use, population (both inhabitants and visitors), and wastewater treatments. Land use in 1956 was determined by performing a supervised classification of orthoimagery generated from the American Army Map Service flights from January 1956 to November 1957, available at the Spanish National Center for Geographic Information (CNIG) (https://www.ign.es/web/en/ign/portal/qsm-cnig).

The hydro-chemical module was fed with nutrient deposition data from measurements of the gaseous, particulate, and dissolved-in-rain concentration of the main chemical forms of N and P (ammonia, nitrogen oxides, nitric acid vapor, particulate, and dissolved ammonium nitrate, phosphate, and total phosphorus), performed for the period June 2016–December 2017, following Garcia-Gomez, Izquieta-Rojano^76^. Measurement plots were located at M01 (lower-altitude area, with human presence) and M02 (upper area, with occasional human presence). The results were extrapolated to the entire catchment, considering altitude, sub-basin, land use, and precipitation. Atmospheric inputs were introduced with daily resolution in the hydro-chemical module as (1) a contribution to the concentration of inorganic N forms in each sub-basin runoff and (2) direct inputs of inorganic N and P to the lake.

### Sediment traps and sediment record

Cylindrical sediment traps (Hydro-Bios Saarso type) were installed at three depths (6, 14, and 40 m) in the deepest area of the western sub-basin. Several surface sediment cores were obtained at the deepest area of the eastern sub-basin using a Glew-type gravity corer and were sliced at 2 mm intervals. An age model was obtained using Pb-210 following Sanchez-Cabeza, Masque^77^. Subsamples from each core slice or trap material samples were analyzed to determine: TP (segmented continuous microflow Autoanalizer AA3); TN and total organic carbon (TOC) (high-temperature catalytic combustion by Skalar Primacs SNC Analyzer); ẟ^15^N/ẟ^13^C (Flash Dynamic Combustion method by Thermo Scientific FlashEA1112 Thermo-Finnigan Nitrogen and Carbon analyzer with DELTA plus (Finnigan MAT) isotope ratio mass spectrometer); and pigments (high-performance liquid chromatography with diode array detection (HPLC 1200—G1315D, Agilent Technologies), previous lyophilization of sediment subsamples and extraction with acetone:methanol:water (80: 15: 5), using trans-β-Apo-8′-carotenal as an internal standard solution, sonication, and centrifugation). Furthermore, diatom clean frustules and chrysophyte cyst suspensions were obtained by oxidizing organic matter with hot hydrogen peroxide 30% v/v, and microscopic slides were mounted using a refractive resin (Naphrax^®^). Taxa were identified under 1000 × light microscopy (LM) with a 100X/1.4 Zeiss Pan-Apochromat objective mounted in a Zeiss scope-A1 microscope equipped with Differential Interference Contrast (Nomarski) optics and dual oil immersion).

### Ecosystem regime shift

The Lagrange multiplier test for conditional heteroscedasticity^28^ was used to determine whether the lake ecosystem could be in a critical transition. This test consists of (1) establishing whether there is an interannual trend in the series and, if so, detrending it; (2) fitting a time series model (Autoregressive integrated moving average, ARIMA) to the series without trend; (3) calculating the squared residuals of the series based on the optimized model; (4) regressing the squared residuals on themselves lagged one-time step; and (5) checking the slope between the two residuals: if the slope is 0 or negative, there is no conditional heteroscedasticity; if the slope is positive, the correlation coefficient of the regression is multiplied by the sample size (n); (6) finally, the value obtained in the previous step is compared with a χ^2^-distribution with one degree of freedom.

### Supplementary Information

Supplementary Information.

## Data Availability

The data that support the findings are available in Dryad at "Lake Sanabria ecosystem shift (1986–2019)", 10.5061/dryad.3j9kd51q2
