# Supplementary material for: Reduced precipitation can induce ecosystem regime shifts in lakes by increasing internal nutrient recycling
Source: Sci Rep. 2024 May 30;14:12408. doi: 10.1038/s41598-024-62810-9 (PMC11137141; doi:10.1038/s41598-024-62810-9)
Supplement: Supplementary file 1 — Supplementary Information. [file 41598_2024_62810_MOESM1_ESM.pdf]

SUPPLEMENTARY INFORMATION

Reduced precipitation can induce ecosystem regime shifts in lakes by increasing internal nutrient recycling

Jordi Catalan[1,2*], Agustín P. Monteoliva[3], José Carlos Vega[4], Almudena Domínguez[5], Ana I. Negro[6], Rocío Alonso[7], Blas Valero Garcés[8], Meritxell Batalla[2], Héctor García-Gómez[7], Manel Leira[9], Carlos Nuño Sánchez[5], José Pahissa[2], María Peg[5], Sergi Pla-Rabés[2,10], Neftalí Roblas[5], José Luis Vargas[5], Manuel Toro[5]

[1]CSIC, Bellaterra, Barcelona, Spain
[2]CREAF, Cerdanyola del Vallés, Barcelona, Spain
[3]ECOHYDROS, Camargo, Cantabria, Spain
[4]Laboratorio de Limnología, Parque Natural del Lago de Sanabria y Alrededores, Rabanillo-Galende, Zamora, Spain.
[5]Centre for Hydrographic Studies, CEDEX, Madrid, Spain.
[6]Area of Ecology, Faculty of Biology, University of Salamanca, Spain
[7]Ecotoxicology of Air Pollution, Environment Department, CIEMAT, Madrid, Spain
[8]Instituto Pirenaico de Ecología, CSIC, Zaragoza, Spain.
[9]Department of Functional Biology, University of Santiago de Compostela, Santiago de Compostela, Spain
[10]Unitat Ecologia, BABVE, Universitat Autònoma de Barcelona, Cerdanyola del Vallés, Barcelona, Spain

***corresponding author**:
J. Catalan, CREAF, Campus UAB, Edifici C, Cerdanyola del Vallés, E-080193, Barcelona, Spain. Email: catalan@ceab.csic.es

CONTENT

Material and Methods Extended (including Table S1)

Fig. S1. Map of Lake Sanabria watershed with place names.

Fig. S2. Lake Sanabria bathymetric map.

Fig. S3. Water column distribution of the phytoplankton groups during 2015-2017.

Fig. S4. Diatom bloom development during 2016-2017.

Fig. S5 Nutrient concentrations in the Tera River before discharging in L. Sanabria.

Fig. S6. Enhanced recycling indicators

Fig. S7. Sediment trap C/N seasonal changes

Fig. S8. Oxygen trends below 30 m depth in Lake Sanabria.

Fig. S9. Depth-age model for Lake Sanabria sediment core.

Fig. S10. Phytoplankton comparison between 1987-89 and 2015-17

Table S1. Data sources and parameters used in the MapShed model

Fig. S11. River Tera inlet comparison between model and measurements

Table S2. Catchment nutrient source model performance

Appendix S1. Simple model of the flow of a limiting nutrient through the lake system.

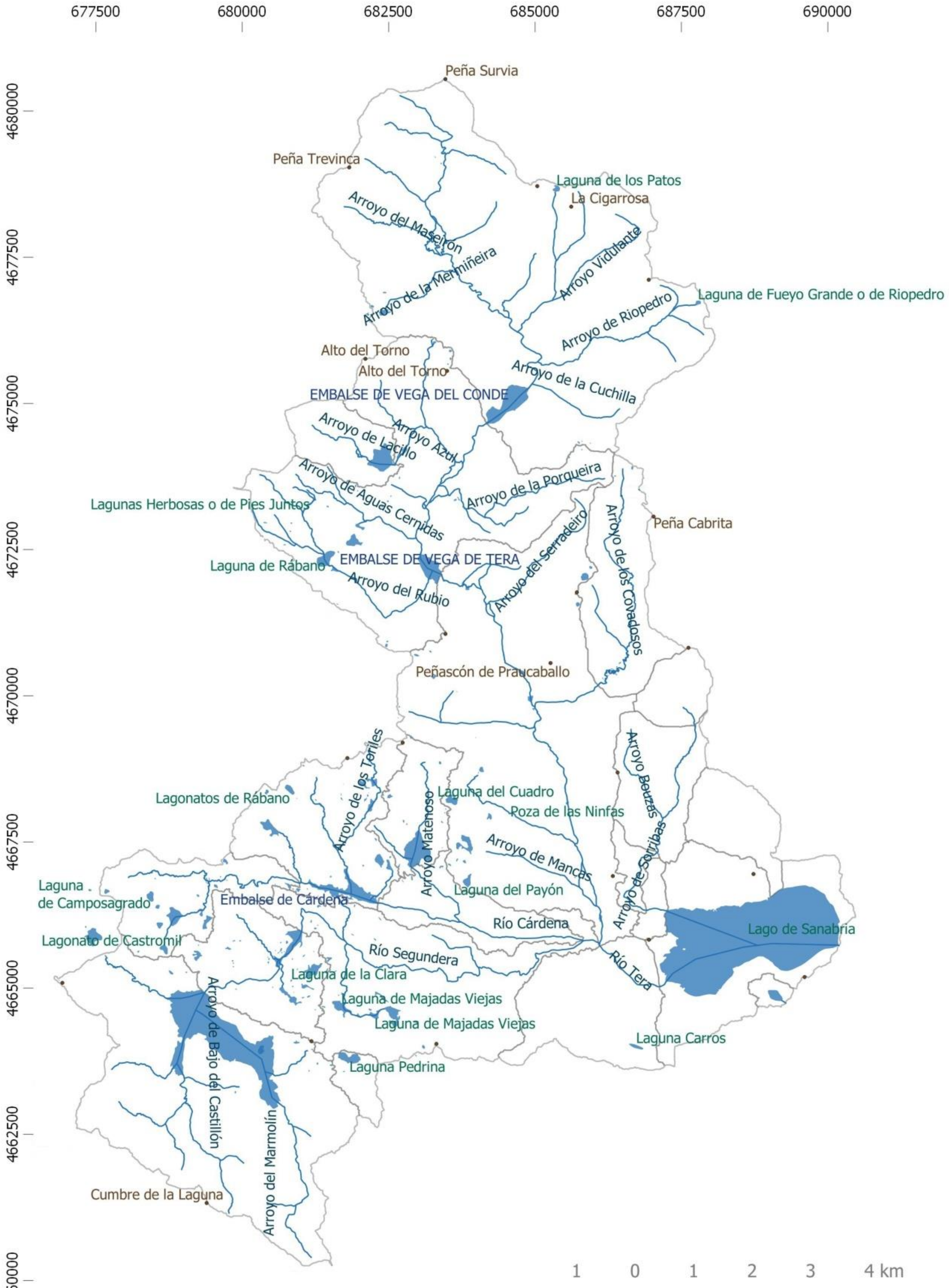

Fig. S1. Map of Lake Sanabria Lake watershed. Cartography extracted from the National Topographic Base of the National Geographic Institute (IGN) and limit of the sub-basins (in gray) by Ecohydros S.L. Reference system: WGS 84 / UTM zone 29N.

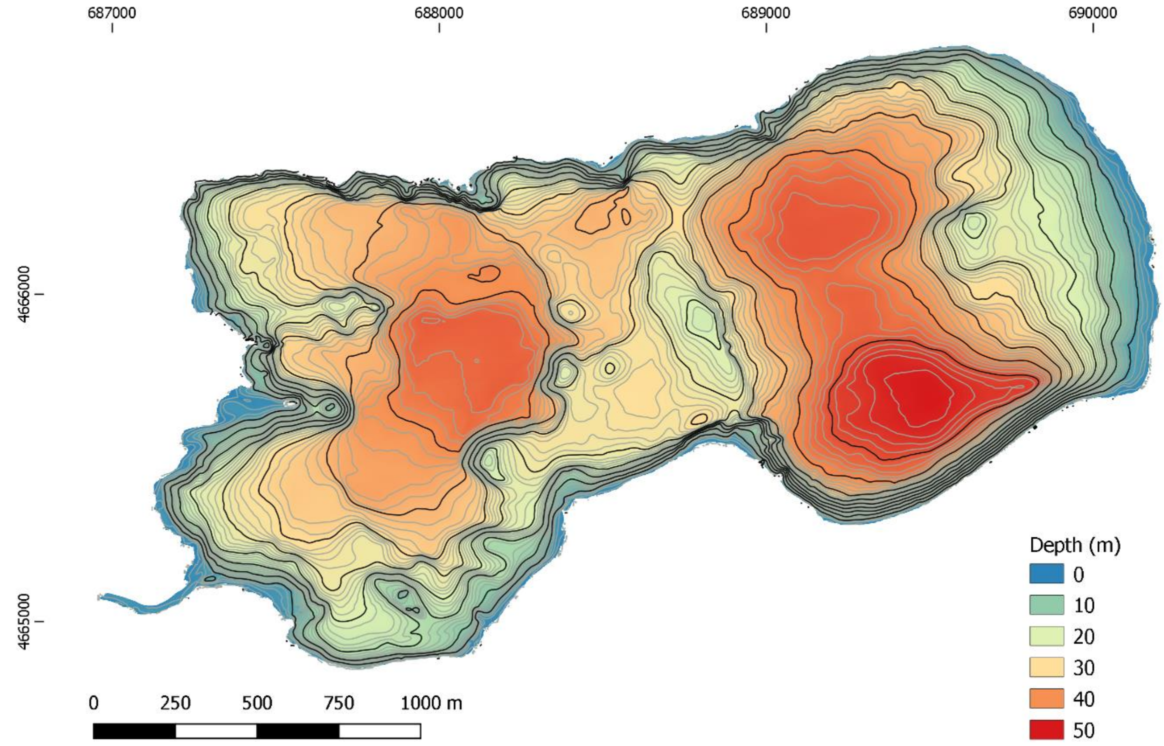

Fig. S2. Lake Sanabria bathymetry developed by Ecohydros Ltd for this study. Technical details can be found in the 2018 report "Adquisición de modelo batimétrico digital del lago de Sanabria (Zamora). Confederación Hidrográfica del Duero, 14 pp. (available in Spanish only)."

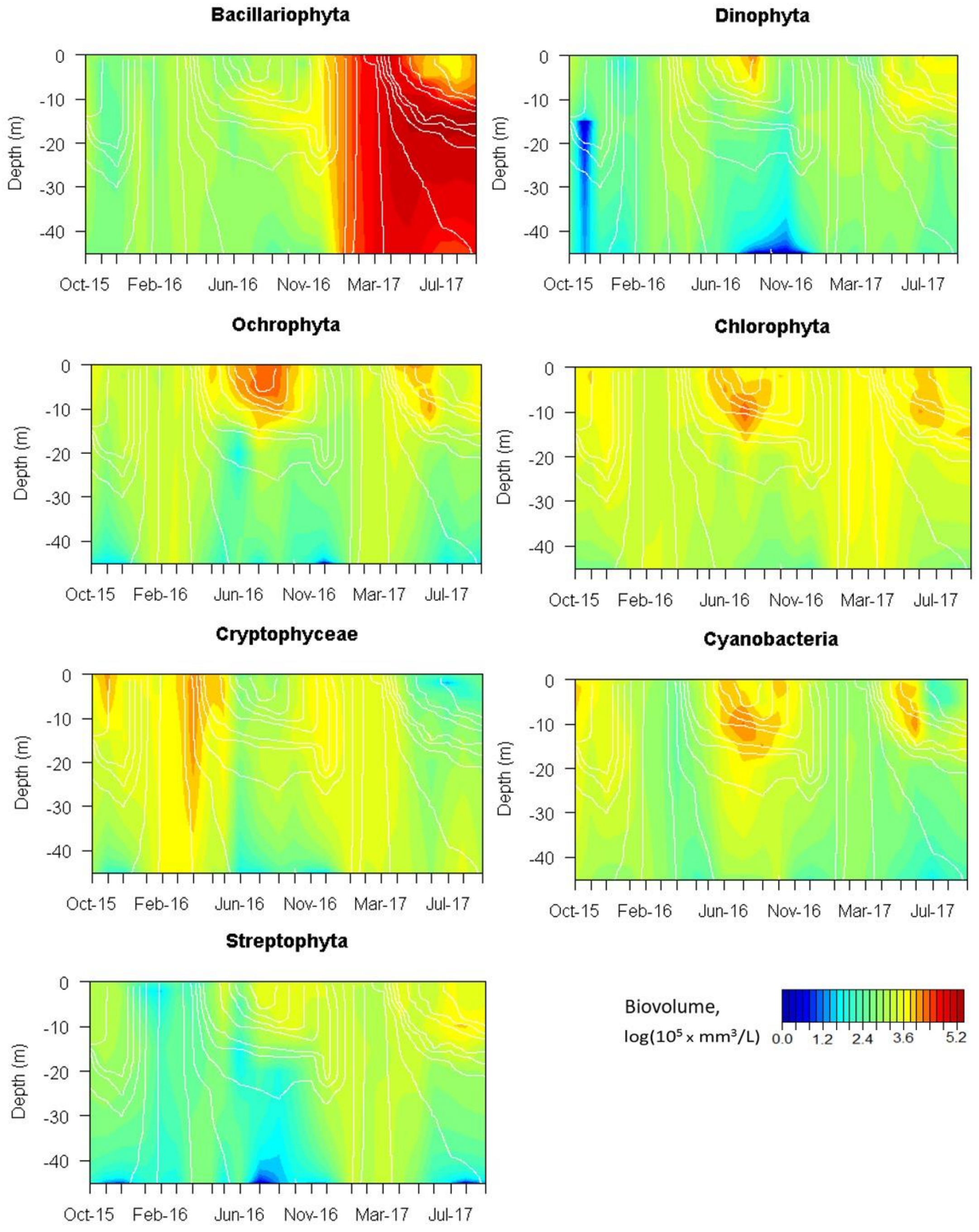

Fig. S3. Biovolume distribution of the main phytoplankton groups before and during the *Asterionella ralfsii* var *americana* bloom. This species mostly accounted for the high Bacillariophyta values.

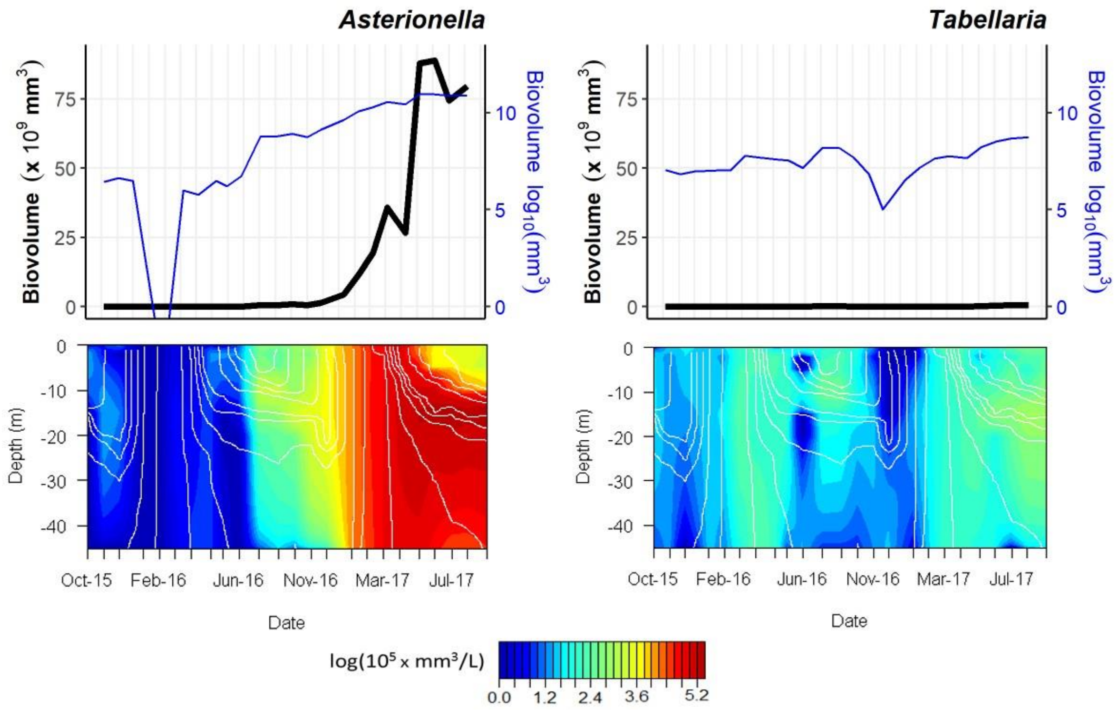

Fig. S4. Changes in *Asterionella ralfsii* var *americana* and *Tabellaria flocculosa* across the water column during the bloom of *Asterionella.* The upper panels indicate the integrated biovolume across the entire lake (mm^3 within the lake). The *Asterionella* growth started during 2016's autumn deepening of the thermocline, developed during the mixing period, and lasted during the whole following stratification period but mostly relegated to metalimnion and upper hypolimnion. In contrast, 2013's *Tabellaria* bloom reached the water column surface and produced some floating scum.

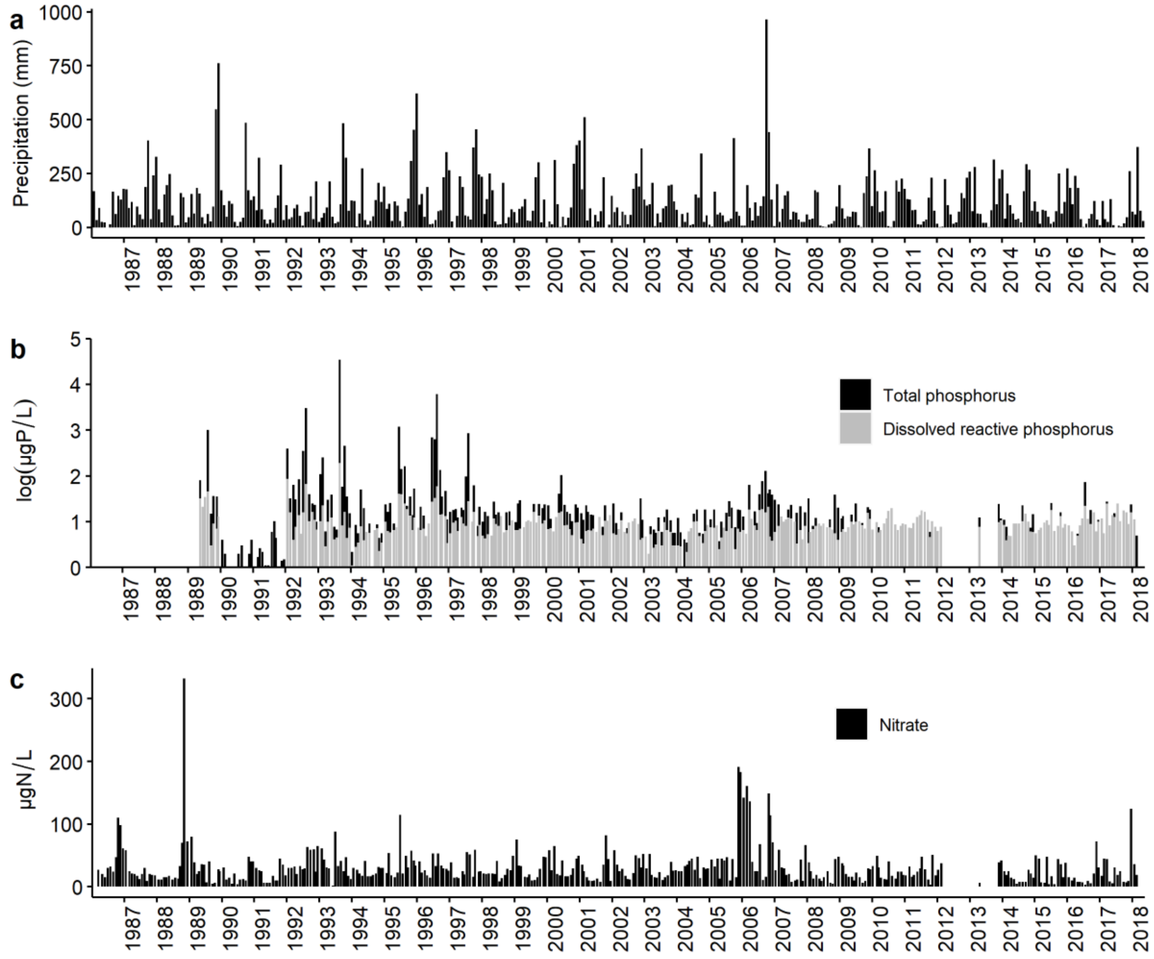

Fig. S5. Monthly precipitation values in M02 station (a), and phosphorus (b) and nitrate (c) concentration in the Tera River before discharging in Lake Sanabria (station I02, Fig. 1). The precipitation data is the same as in Fig. 5 of the main text but shown with different temporal window. The high nitrate values during 2006 and 2007 correspond to a large fire in the catchment.

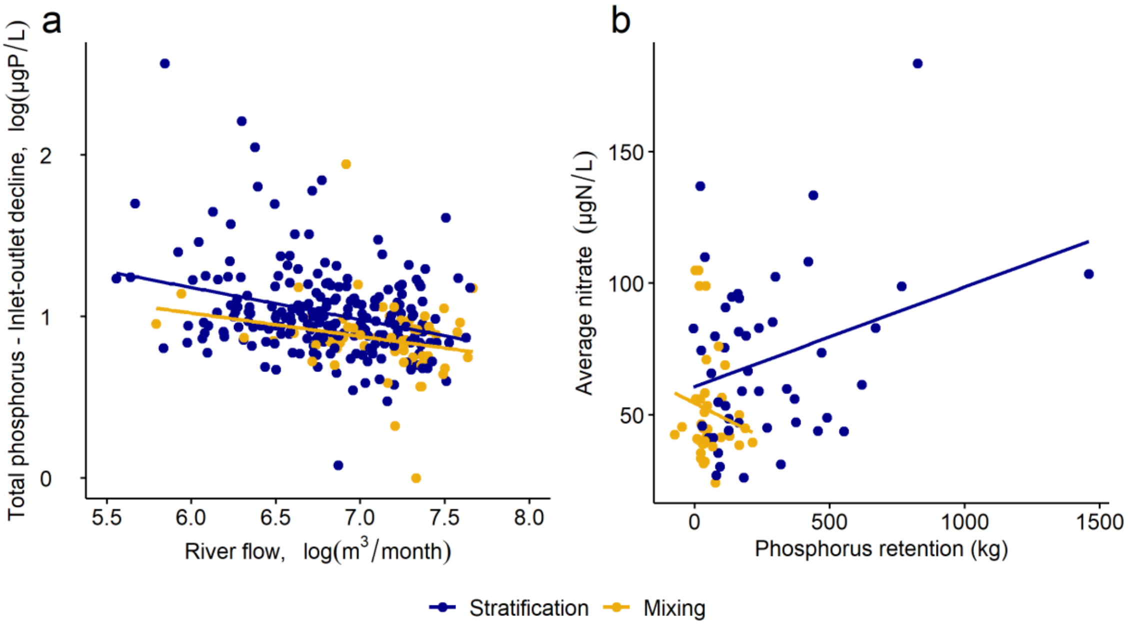

Fig. S6. Enhanced recycling indicators. (a) The relationship between river flow and the total phosphorus difference between the main lake inlet (Tera river) and the outlet indicates retention efficiency (Multivariate regression, R^2_{adj} = 0.13, P= 4.5 x 10^{-10}, n=295). (b) Relationship between phosphorus retention and average nitrate in the water column (Multivariate regression, R^2_{adj} = 0.15, P < 0.0005, n=85). Multivariate regression accounted for different coefficients for stratification and mixing periods.

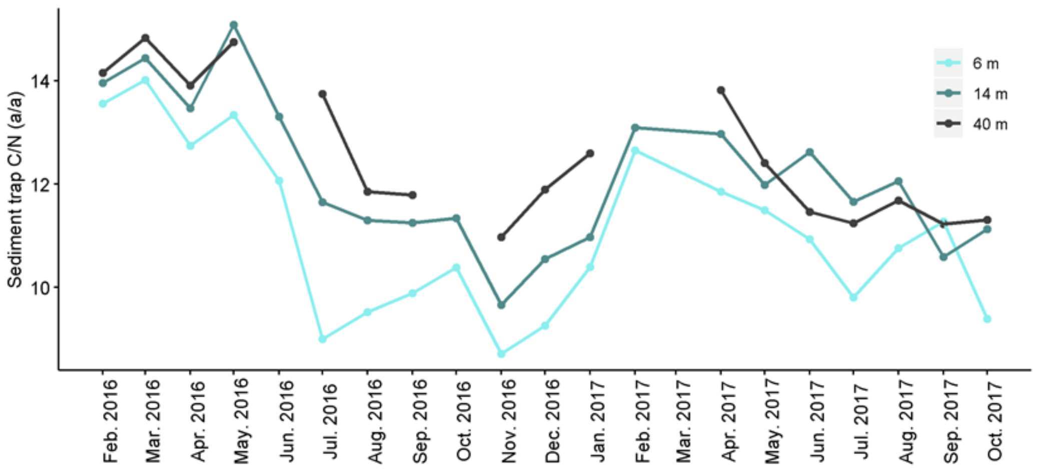

Fig. S7. Ratio between carbon and nitrogen in the organic matter from three sediment traps located in the D02 station of Lake Sanabria from February 2016 to October 2017. They show nitrogen impoverishment (recycling) as the organic matter sinks through the water column (ANOVA, P=0.007, n=57). The seasonal fluctuations are due to the relative contribution of allochthonous organic matter, with higher C/N, and lake primary production matter, which is relatively richer in nitrogen.

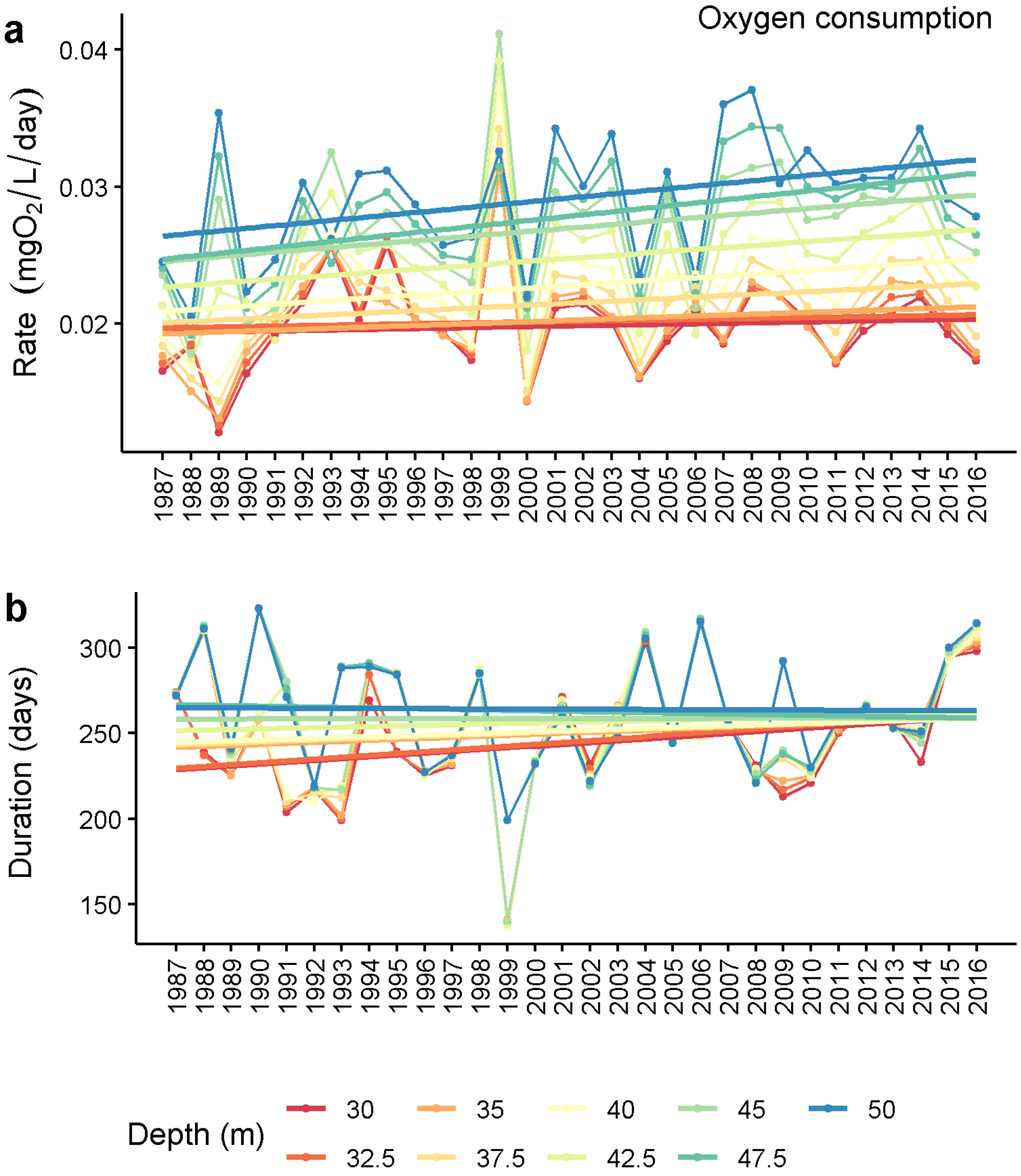

Fig. S8. Oxygen decline trends during stratification below 30 m depth in Lake Sanabria. (a) Oxygen consumption rates. Only data from 47.5 m depth show significant Mann-Kendal test (P=0.027, n=30). (b) Duration of the period of continued oxygen decline.

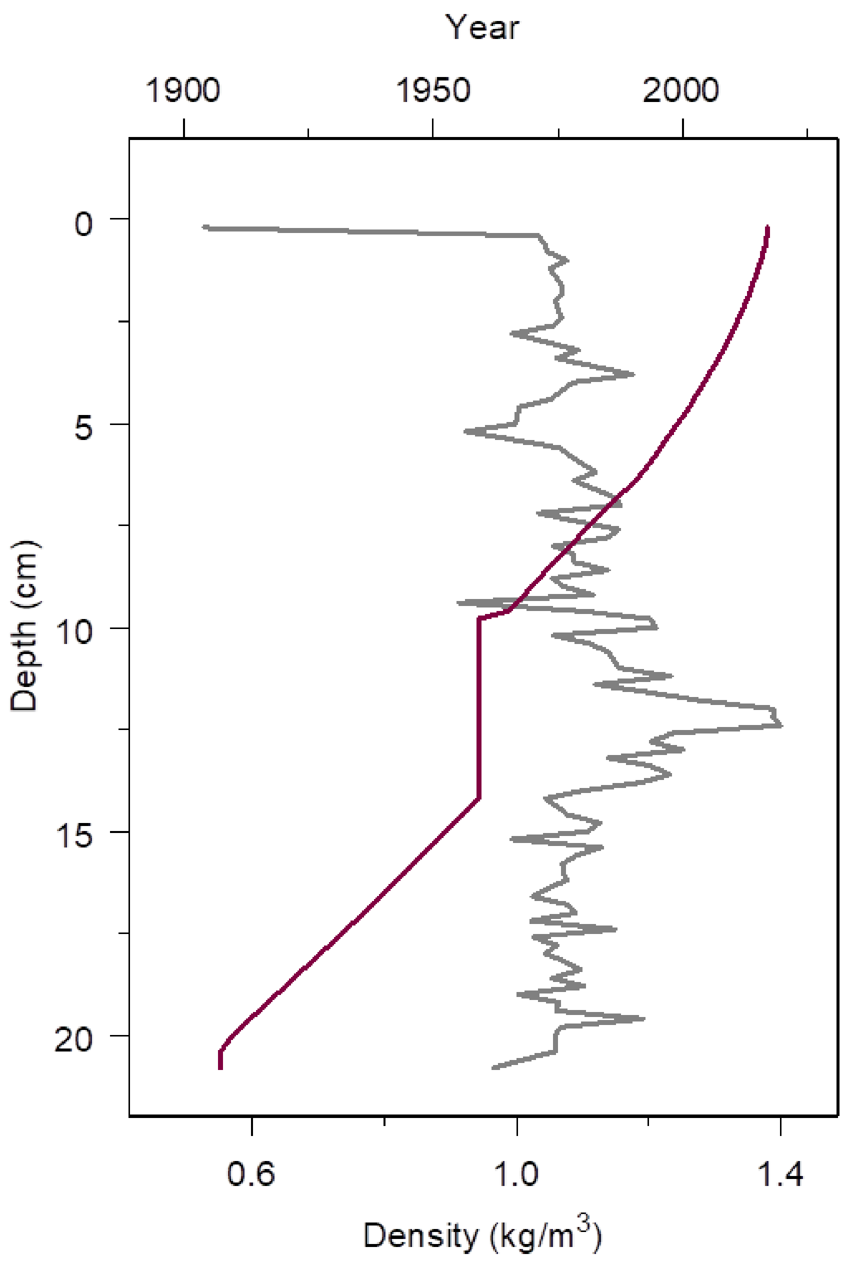

Fig. S9. Depth-age model (red line) of the Lake Sanabria sediment core according to Pb-210 dating (performed by P. Masqué, Edith Cowan University, Australia). The increase in sediment density (grey line) between 10-14 cm indicates the flooding during the Vega de Tera dam collapse on 9 January 1959.

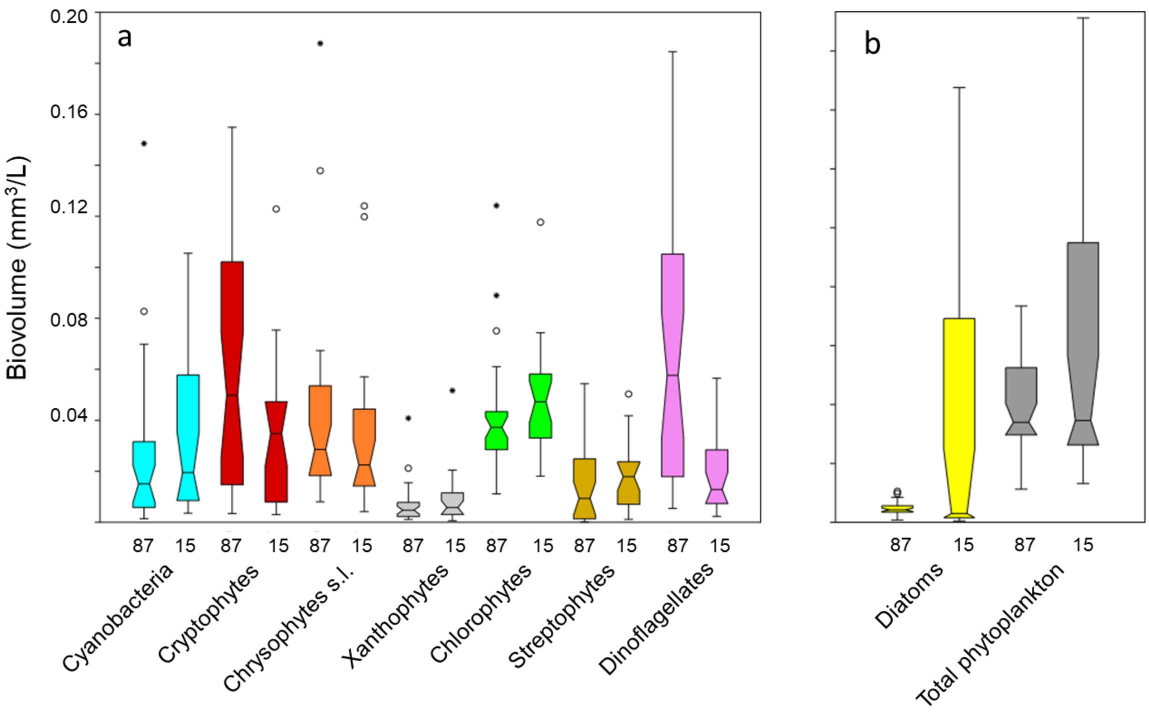

Fig. S10. Comparison of phytoplankton group biovolume monthly observations between 1987-1989 and 2015-17. (a) All groups without diatoms. (b) Diatoms and total phytoplankton biovolume. At lower taxonomic levels, *Cryptomonas* spp., athecate dinoflagellates, *Chlorella* sp., *Synechococcus capitatus* were significantly more abundant in 1987-1989 observations, while *Anathece* spp., *Pediastrum privum*, *Oocystis* cf. *submarina*, and filamentous desmids, were more abundant in 2015-2017. Integrated samples from 0 to 15 m depth were collected at the D02 station.

Table S1. Data sources and critical parameters applied in the MapShed model

Data or parameter	Source/method	Units	Value
**Soils**			
Soil-type polygon layer (soil map)	Institute of Natural Resources and Agrobiology of Salamanca (IRNASA-CSIC)	-	-
The water-holding capacity of the soil	Evans & Corradini, 2016	cm	7
Dominant soil hydrologic group class	Evans & Corradini, 2016	-	A
**Erosion (USLE)**			
Land use/cover map	Segmentation and supervised classification on PNOA aerial orthophoto (2014)	-	-
Curve hydrologic number (CN)	Evans & Corradini, 2016 / Estimation from soil and land use maps.	-	0 - 72
Erodibility (or "K" factor)	The method in Gisbert Blanquer et al. (2012) applied to the soil map.	-	0.19 – 0.29 (0.05 for bare rock class)
Slope-Length (LS) Factor	Evans & Corradini, 2016 / Option using the algorithm contained within the ArcView "Terrain Analysis." Estimation by land use class within sub-basin.	-	0 - 10
Crop management (C) and Erosion control practice (P) factors	Evans & Corradini, 2016 / Default values by land use class.	-	0 - 1
**Water balance**			
Evapotranspiration coefficients	Evans & Corradini, 2016 / ET coefficients are assigned by land use/cover type and are area-weighted to determine average values for each month of the year.	-	0.3 - 1
ET adjustment factor	Used to calibrate the model in the control points with flow data.	%	40 - 50
Groundwater seepage coefficient	The fraction of infiltrated water lost to an underlying aquifer or deep saturated zone that was used to calibrate the model in the control points with flow data.	-	0 - 1
Groundwater recession coefficient	Modulates the peak flow/base flow relationship in hydrographs. Used to calibrate the model in the control points with flow data.	-	0 - 1
Unsaturated Available Water-Holding Capacity	An area-weighted value for all the soil mapping units in each watershed is automatically calculated	cm	10 - 30
**Nutrients**			
Dissolved runoff coefficients for nitrogen	Estimates for each sub-basin and land use (7 types) were based on atmospheric modeling analysis and actual wet and dry deposition measurements. It was assumed that the concentration in the wet deposition was maintained in the runoff and dry deposition accumulated in each sub-basin until the next rainfall event.	µg_N/L	10 - 500
Dissolved runoff coefficients for phosphorus	Estimates for each sub-basin and land use (7 types) were based on atmospheric modeling analysis and actual wet and dry deposition measurements. It was assumed that the concentration in the wet deposition was maintained in the runoff and dry deposition accumulated in each sub-basin until the next rainfall event.	µg_P/L	1 - 25
Nitrogen in groundwater	Average TN values in lixiviate samples in 5 points representative of different land use, 10 to 18 samples over 18 months.	µg_N/L	200 - 450

Phosphorus in groundwater	Average TP values in lixiviate samples in 5 points representative of different land use, along 10 to 18 months.	µg_P/L	15 - 25
Nitrogen in sediment	Area-weighted value for all the soil mapping units in each watershed of the TN values in soil samples (ITACyL).	mg_N/kg	830 - 1600
Phosphorus in sediment	Area-weighted value for all the soil mapping units in each watershed of the TP values in soil samples (ITACyL).	mg_P_P_2O_5/kg	17 - 38
Reservoir nutrient retention coefficients	Evans & Corradini, 2016 / Allow accounting for (i.e., approximate) the pollutant attenuating effect of reservoirs	% TP	29
		% TN	12
		% TSS	84
**Livestock contribution**			
Number of cows	Regional livestock census	-	2687
Number of sheep	Regional livestock census	-	2449
Average weight per cow	Regional livestock census	kg	360
Average weight per sheep	Regional livestock census	kg	50
Nitrogen daily load for cows	Evans & Corradini, 2016 / Typical values	g_N/kg	0.31
Phosphorus daily load for cows	Evans & Corradini, 2016 / Typical values	g_N/kg	0.10
Nitrogen daily load for sheep	Evans & Corradini, 2016 / Typical values	g_N/kg	0.37
Phosphorus daily load for sheep	Evans & Corradini, 2016 / Typical values	g_N/kg	0.09
Grazing period	Transhumance behavior. Only from June to October in the watershed.	months/year	5
Time spent in streams	Evans & Corradini, 2016 / Typical values.	%	5
Nitrogen loss rate	Soupir et al., 2006 / Amount of N delivered (i.e., "lost") to surface water.	%	5
Phosphorus loss rate	Soupir et al., 2006 / Amount of P delivered (i.e., "lost") to surface water.	%	7
**Wastewater (WWTP effluents)**			
Ribadelago WWTP effluent	Discharge and nutrient concentration data during 18 months.		
Camping and beach area "Los Robles"	Number of visitors estimated from an automatic vehicle counting system.	people/month	7000 - 100000
	Number of visitors equaling one habitant equivalent (the use of the area takes only a portion of the day)	people	12
	Service period: Mid-June to mid-September	months	3

Evans, B. M., & Corradini, K. J. (2016). *MapShed Version 1.5 Users Guide. Prepared for Penn State Institutes of Energy and the Environment*. PA: University Park.

Gisbert Blanquer, J. M., Ibáñez Asensio, S., & Moreno Ramón, H. (2012). *El factor K de la ecuación universal de pérdidas de suelo (USLE).* . Valencia, Spain: Escuela Técnica Superior de Ingeniería Agronómica y del Medio Natural. Universidad Politécnica de Valencia.

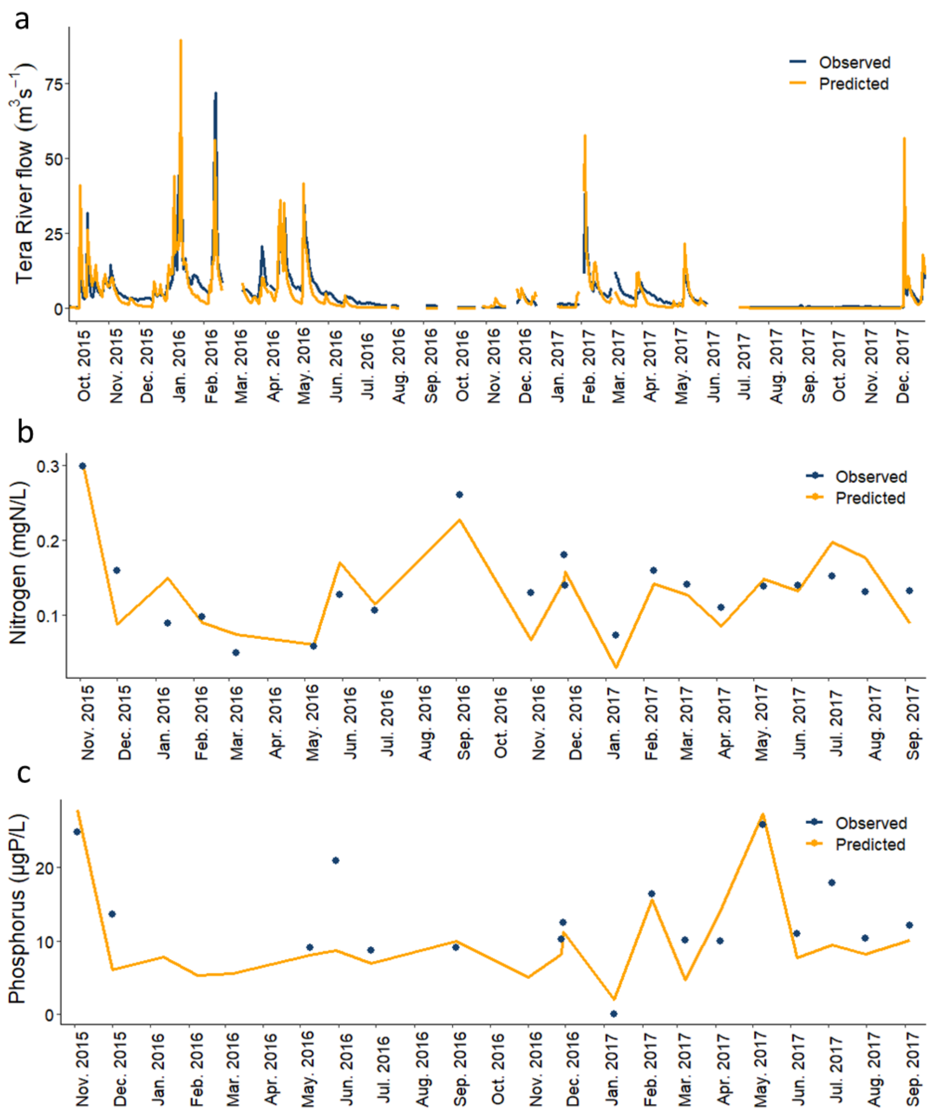

Fig. S11. Comparison between modeled and measured values in the river Tera inlet. (a) Flow. (b) Total nitrogen. (c) Total phosphorus. See Table S2 for comparative statistics.

Table S2. Comparison between measured and modeled data at the Tera River inlet for the period 2015-2017 in Fig. S11.

	R2	RMSE	RSR	NSE	PBIAS
Discharge (m^3/s)	0.61	4.59	0.43	0.61	16.32 %
Total phosphorus (µ/L)	0.64	4.58	0.73	0.64	-4.03 %
Total nitrogen (mg/L)	0.68	0.04	0.61	0.61	3.35 %

R2 – R-squared. RMSE – Root mean squared error. RSR- Ratio of the root mean squared error to the standard of the measured data. NSE - Nash-Sutcliffe efficiency. PBIAS – percent bias.

Appendix S1

Simple model of the flow of a limiting nutrient through the lake system.

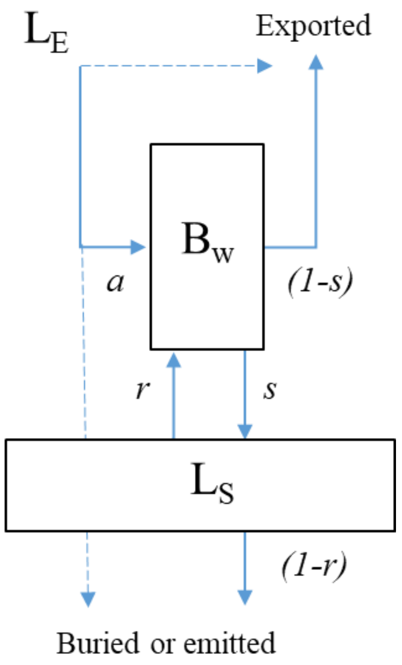

L_E – Total external loading of the nutrient to the lake.

B_W – Amount of the nutrient in the lake biomass.

L_S – Load of the nutrient in top functional sediment.

a – Fraction of the external loading that is successfully incorporated in living biomass. The rest is either exported downstream, permanently buried in the sediment, or emitted to the atmosphere.

s – Fraction of living biomass incorporated into the functional sediment. The rest is exported downstream.

r – Fraction of the nutrient sediment load that returns to the water column and is assimilated in living biomass. The rest is permanently buried or emitted into the atmosphere.

In a steady state of the system at the appropriate time scale:

$$\frac{d\mathrm{B}_W}{dt} = aL_E + rL_S - B_W = 0 \qquad (1)$$

$$\frac{d\mathrm{L}_S}{dt} = sB_W - L_S = 0 \qquad (2)$$

and thus

$$B_W = \frac{a\,L_E}{1-r\,s} \qquad (3)$$

The condition for an increase in B_W in a new steady state is:

$$B_{W1} > B_{Wo} \qquad (4)$$

$$\frac{a_1\,L_{E1}}{1-r_1 s_1} > \frac{a_0\,L_{E0}}{1-r_0 s_0} \qquad (5)$$

The fraction (s) of living biomass that sinks to the sediments is close to 1 if the water residence time (t_r) is sufficiently larger than the characteristic sinking time of biomass (t_s), which is the case for most lakes, except when the high inflow compared to volume result in $t_r << 1$ year.

$$s \sim 1 - \frac{t_s}{t_r} \qquad (6)$$

t_s is proportional to the quotient between the depth at which contact between water and sediment increases exponentially (ca. 30 m depth in L. Sanabria) and a characteristic sinking velocity, which may vary between 0.14 m d^{-1} for small picoplankton cells to 0.8 m d^{-1} for large diatoms [1]. Therefore, in the case of L. Sanabria, $t_s \approx 0.1$- 0.5 years. On the other hand, t_r fluctuates between about 4.5 years when precipitation is high (e.g., 2000 L m^{-2} y^{-1}) and transpiration low (meadows predominate) and about 14.5 years when precipitation is low (e.g., 800 L m^{-2} y^{-1}) and transpiration high (forest predominate). Even the large difference in t_r, s will range between 0.89 and 0.98 because $t_r >> t_s$.

If s can be assumed close to 1, then equation (5) becomes

$$\frac{a_1\, L_{E1}}{1 - r_1} > \frac{a_0\, L_{E0}}{1 - r_0} \qquad (7)$$

and, consequently, if the external load declines ($L_{E1} < L_{E0}$) but water column biomass increases ($B_{W1} > B_{Wo}$) then

$$\frac{L_{E1}}{L_{Eo}} \frac{a_1}{1 - r_1} > \frac{a_0}{1 - r_0} \qquad (8)$$

the factor that considers the efficiency in incorporating external loading into biomass and sediment recycling has to increase, at least more than the decline in external loading.

1. Reynolds CS. *The ecology of phytoplankton*. Cambridge University Press (2006).
